# A GSTP1-mediated lactic acid signaling promotes tumorigenesis through the PPP oxidative branch

**DOI:** 10.1038/s41419-023-05998-4

**Published:** 2023-07-25

**Authors:** Yandi Sun, Qian He, Jingjia Li, Ze Yang, Mashaal Ahmad, Yindan Lin, Di Wu, Lei Zheng, Jiangtao Li, Ben Wang, Chitty Chen, Yue Hu, Heng Luo, Yan Luo

**Affiliations:** 1 Cancer Institute, The Second Affiliated Hospital, Zhejiang University, Hangzhou, Zhejiang China; 2 Institute of Translational Medicine, School of Medicine, Zhejiang University, Hangzhou, Zhejiang China; 3 Department of Biochemistry & Cancer Medicine, International Institutes of Medicine, the Fourth Affiliated Hospital of Zhejiang University School of Medicine, Yiwu, Zhejiang China; 4 Department of Biochemistry and Molecular Biology, School of Medicine, Zhejiang University, 866 Yu Hang Tang Road, Hangzhou, Zhejiang China; 5 Department of General Surgery, the Second Affiliated Hospital, Zhejiang University School of Medicine, Hangzhou, Zhejiang China; 6 Departments of Oncology and Surgery, the Pancreatic Cancer Center of Excellence Program, the Johns Hopkins University School of Medicine, Baltimore, MD USA; 7 Department of Research and Development, SysDiagno Biotech, Nanjing, 211800 Jiangsu Province China; 8 Department of Breast Surgery, the Second Affiliated Hospital, Zhejiang University School of Medicine, Hangzhou, Zhejiang China; 9 State Key Laboratory of Functions and Applications of Medicinal Plants, Guizhou Medical University, Guiyang, Guizhou China; 10 The Key Laboratory of Chemistry for Natural Products of Guizhou Province and Chinese Academy of Sciences, Guiyang, Guizhou China

**Keywords:** Phosphorylation, Breast cancer

## Abstract

Lactic acidosis is a feature of solid tumors and plays fundamental role(s) rendering cancer cells to adapt to diverse metabolic stresses, but the mechanism underlying its roles in redox homeostasis remains elusive. Here we show that G6PD is phosphorylated at tyrosine 249/322 by the SRC through the formation of a GSTP1-G6PD-SRC complex. Lactic acid attenuates this formation and the phosphorylation of G6PD by non-covalently binding with GSTP1. Furthermore, lactic acid increases the activity of G6PD and facilitates the PPP (NADPH production) through its sensor GSTP1, thereby exhibiting resistance to reactive oxygen species when glucose is scarce. Abrogating a GSTP1-mediated lactic acid signaling showed attenuated tumor growth and reduced resistance to ROS in breast cancer cells. Importantly, positive correlations between immuno-enriched SRC protein and G6PD Y249/322 phosphorylation specifically manifest in ER/PR positive or HER negative types of breast cancer. Taken together, these results suggest that GSTP1 plays a key role in tumor development by functioning as a novel lactate sensor.

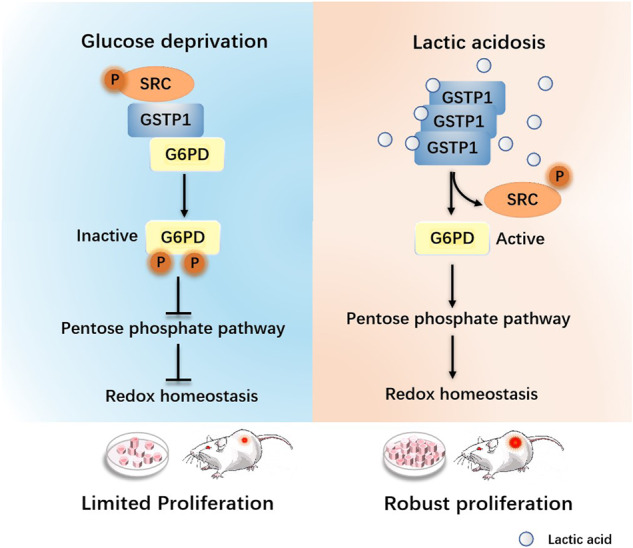

## Introduction

Altered metabolism is a key feature of tumor cells [1–4]. In early 1920’s, Otto Warburg proposed a unique metabolic model for tumor cells [5]: they generate energy even under aerobic conditions by glycolysis to produce enough ATP to satisfy the requirements of proliferative cells [6–8]. For these cells to proliferate, a glycolytic flux needs to be constrained to allow a higher percentage of intermediates to be diverted to biosynthesis, e.g., availing G6P (glucose-6-phosphate) to the pentose phosphate pathway (PPP), thus an exquisite balance between fluxes of glycolysis and PPP that benefits tumor development. The pentose phosphate pathway is the major path to produce NADPH via two key enzymes G6PD (Glucose-6-phosphate Dehydrogenase) and PGD (6-phosphogluconate dehydrogenase), which provides the reducing power to sustain redox balance. In addition, ribose-5-phosphate as a key metabolic intermediate of the pentose phosphate pathway is the precursor for DNA/RNA synthesis, vital for the growth of cancer cells [9–11].

Due to aerobic glycolysis and deformed angiogenesis in solid tumors, nutrients such as glucose and oxygen are very scarce, especially in the inner part. Thus, glucose deprivation and high levels of lactic acid eventually form the tumor metabolic microenvironment. Reportedly, glucose deprivation boosts up the reactive oxygen species and causes the death of cancer cells through inhibiting glycolysis and the pentose phosphate pathway [12], and lactic acid is known to promote NADPH production and maintains redox balance [13]. However, an involvement of the pentose phosphate pathway and mechanistic description of lactic acid-mediated up-regulation of the major reducing power production path remain unaddressed.

Lactic acid is the end product of aerobic glycolysis [14, 15]. This metabolite is quite often dismissed as a waste product, but recent evidence points to its roles in tumorigenesis beyond serving a fuel molecule [16, 17]. For example, lactic acid helps cancer cells to acquire growth advantage in metabolic stress via a feedback mechanism modulating the activities or expression of key glycolytic enzymes such as hexokinase and PKM2 [18, 19]. Indeed, cancer cells cultured with limiting glucose sustain proliferation only under lactic acidosis, which feeds back to reduce the glycolytic glucose consumption. Lactate also increases the expression of the glutaminase transporter ASCT2 and glutaminase 1 (GLS1) genes, which enhances the TCA cycle flux by augmenting the mitochondrial α-ketoglutarate level [20] in an anaplerotic fashion that would save glucose carbons for biomass building rather than for energetics. Further, lactic acid acts as a signaling molecule to regulate cellular behaviors including metabolism [21–23]. For example, hypoxia-induced lactic acid allows its interaction with NDRG3, which in turn activates the c-Raf/ERK signaling pathway to promote tumorigenic proliferation and angiogenesis [24]. Taken together, these studies indicate that lactic acidosis plays multiple roles in modulating metabolic behaviors of cancer cells; however, the precise mechanisms underlying the roles of lactic acid in cancer development, under diverse metabolic stresses in particular, remain largely elusive.

Glutathione S-transferases (GSTs) have seven classes and their Roman capitals are A, M, P, S, T, O, and Z [25, 26]. GSTs as housekeepers catalyze the glutathione (GSH) conjugating to some electrophilic compounds [27], thus determining its classic role in the detoxification of exogenous substances such as anti-cancer drugs [28]. Reportedly, glutathione S-transferase pi (GSTP1) is highly expressed in breast cancer, colon cancer, and ovarian cancer and is related with the progression of drug-resistant solid tumor [29, 30]. Moreover, GSTP1 is associated with the MAPK pathway and TNF receptor-associated factor 2 (TRAF2) [31] to participate in the cell apoptosis, stress stimuli, cell differentiation and proliferation. For example, GSTP1 can bind to JNK and inhibit its activity and apoptosis [32].

In this study, we investigated the role(s) of a glutathione S-transferase pi (GSTP1)-mediated lactic acid signaling that impacts the pentose phosphate pathway and redox balance, especially under glucose deprivation, emphasizing role(s) of a Lac-GSTP1-G6PD axis in ER/PR positive or HER negative types of breast cancer.

## Results

### Lactic acid sustains the growth of cancer cells under glucose deprivation

It has been reported that the inner pH of solid tumors can reach 6.3 ~ 6.8 in conjunction with glucose deprivation as severe as 1/10th of the normal blood sugar level, and the levels of lactic acid typically range from 3 mM to 20 mM in most solid tumors [33–35]. In extreme, it can reach as high as 40 mM in gastrointestinal cancer. By examining the pH values of media containing 0.5 mM glucose with a titration of lactic acid (0-20 mM), we found that the pH of 20 mM lactic acid was much closer to reported inner pH of solid tumor (Figure S1A). Therefore, this metabolic microenvironment of solid tumors can be recapitulated, and throughout the study we used 0.5 mM glucose in media to recapitulate glucose deprivation and, on top of this, added 20 mM lactic acid for a mimicry of lactic acidosis.

**Fig. 1:**
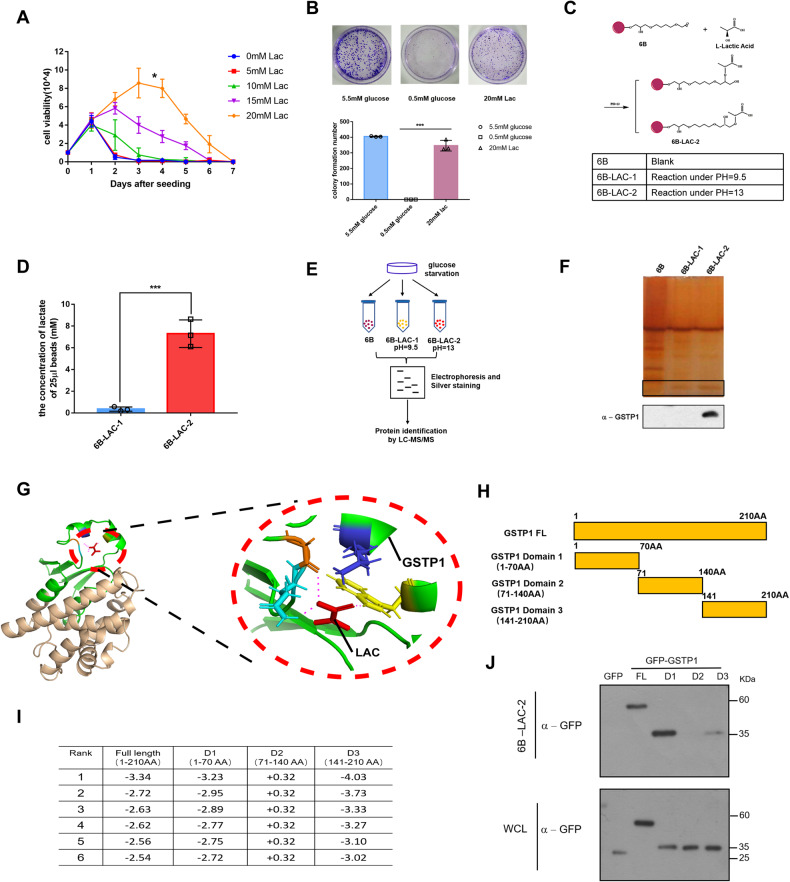
GSTP1 is a novel target for lactic acid. **A** The growth curve of MCF-7 cells cultured in glucose-deprived (0.5 mM) media with variable, as indicated, lactic acid levels. Results are mean ± SD of triplicate experiments. *p (-lac vs 20 mM lac) = 0.0118. **B** Colony formation assay of MCF-7 cells cultured in media containing, as indicated, 5.5 mM glucose, or 0.5 mM glucose supplemented without or with 20 mM lactic acid for 7 days. Numbers of colonies were determined. Results are mean ± SD of triplicate experiments. ***p (0.5 mM glucose vs 20 mM Lac) = 0.0006. **C** A schematic diagram for Epoxy-activated Sepharose 6B that was cross-linked with lactate. Detailed information of two kinds of beads under different pH is shown. **D** The concentration of lactate linking to Epoxy-activated Sepharose 6B (25 µl) was determined at 530 nm by chromogenic reaction. **E** A flow chart of the identification of lactate-binding proteins. **F** Eluted protein profiles from 3 types of resins. Upper panel, silver stain; bottom panel, immuno-blot with anti-GSTP1 antibodies detecting enriched GSTP1 by the resin with coupled ligand (3rd lane). 6B: empty Sepharose 6B beads as control; 6B-Lac-1: beads cross-linked with lactate under pH 9.5; 6B-Lac-2: beads cross-linked with lactate under pH 13. Only the last type of resin harbors coupled ligand. **G** Modeled structure of parts of GSTP1 (PDB ID: 3GUS) with lactate labelled in red, the green area represents parts of GSTP1 involved in lactate binding. **H** A schematic diagram for three regions of GSTP1. **I** Top 6 ranked binding energy values (kcal/mol) are shown. **J** Three regions were overexpressed in MCF-7 cells, enriched by the 6B-LAC-2 beads and eluted fractions analyzed by immuno-blots.

To determine the role(s) of lactic acid in cell proliferation in response to glucose deprivation, we treated cultured human breast cancer cells (MCF-7) in 0.5 mM glucose with incremental lactic acid (0–20 mM). In proliferation analyses that lasted for 7 days, glucose deprived cells lost capability to grow; however, lactic acid especially at 20 mM sustained not only cells’ survival but also, albeit limited, proliferation (Fig. 1A), which was also exhibited in colony formation assays (Fig. 1B). Lactic acid comprises the lactate and proton. Sodium lactate provides lactate and hydrochloric acid at 20 mM was adjusted to pH 6.8 to represent proton. To determine which (lactate or proton) plays a primary role in supporting cell proliferation, we again carried out cell growth analyses. Lactic acid and to a lesser degree lactate supported cell growth, but the proton alone did not (Fig. S1B). These findings prompted a search for potential lactate-binding protein(s) that play critical roles mediating a proliferation-promotion function.

### GSTP1 is a novel target for lactic acid

We reasoned that lactate associated with an effector protein, which might then exert regulatory function(s) to impact metabolic pathways including PPP. Active groups of epoxy-activated sepharose-6B can chemically react with diverse hydroxyl groups under alkaline conditions [36], but for any given ligand an optimal pH should be empirically determined. We thus created two epoxy-activated sepharose-6B resins to which lactate was coupled at either pH9.5 (6B-LAC-1) or pH13 (6B-LAC-2) (Fig. 1C). Chromogenic (Fig. S1C) and spectrophotometer (Fig. 1D) assays, which compared the amounts of lactate coupled to the two types of resins (along with empty resins), indicated that the 6B-LAC-2 resins contained covalently-linked lactate at much higher efficacy.

MCF-7 whole cell lysates were mixed with the resins and subjected to affinity enrichment (Fig. 1E). An eluted fraction from the lactate-coupled 6B-LAC-2 resin specifically enriched Glutathione S-transferase Pi (GSTP1) as an associated protein that was confirmed by immuno-blot (Fig. 1F) after identification by mass spectrometry. Indeed, of potential lactate-binding proteins revealed by quantitative mass spectrometry, the abundance of GSTP1 ranked first (Figure S1D). Reportedly, the dimeric form of GSTP1 was likely to conjugate to the electrophiles [37], and GSTP1 was shown as the 23 kDa monomeric form, the 46-kDa dimeric form and other multimeric forms [38]. The dimeric and the multimeric forms both bind to lactate through Native-page and cross-linking of 0.03125% glutaraldehyde (Fig. S1E, S1F). Hence, the GSTP1-lactate interaction has quite a distinguishable selectivity underscoring a preferred specificity for lactate.

To pinpoint the potential binding domain(s) of GSTP1 with lactate, we predicted the conformation between lactate and GSTP1 (PDB ID: 3GUS) through Autodock tools (Fig. 1G), and mapped the most possible binding region(s). An N-terminus 1-70 region or a C-terminus 141-210 region (Fig. 1H) was each predicted to possess a binding pocket with lactate when analyzed through the Lamrckian GA 4.2 algorithm, which calculates the binding free energy between the lactate and GSTP1. The binding free energy values with the full-length GSTP1 or predictive binding domains were quite negative (the top six shown in Fig. 1I), indicating high potential of interactions. To validate this prediction, we made truncated GSTP1 versions each covering 1/3 of the protein and found that lactate bound to the full-length GSTP1, its N-terminal 1-70 region and to a lesser degree the C-terminal 141-210 region (Fig. 1J). Thus, GSTP1 is a lactate–binding protein, the function of which is probably modulated in response to lactic acid levels that constitute, at least in part, a tumor metabolic micro-environment.

### G6PD is dynamically Tyr-phosphorylated at Y249/Y322 by SRC

**Fig. 2:**
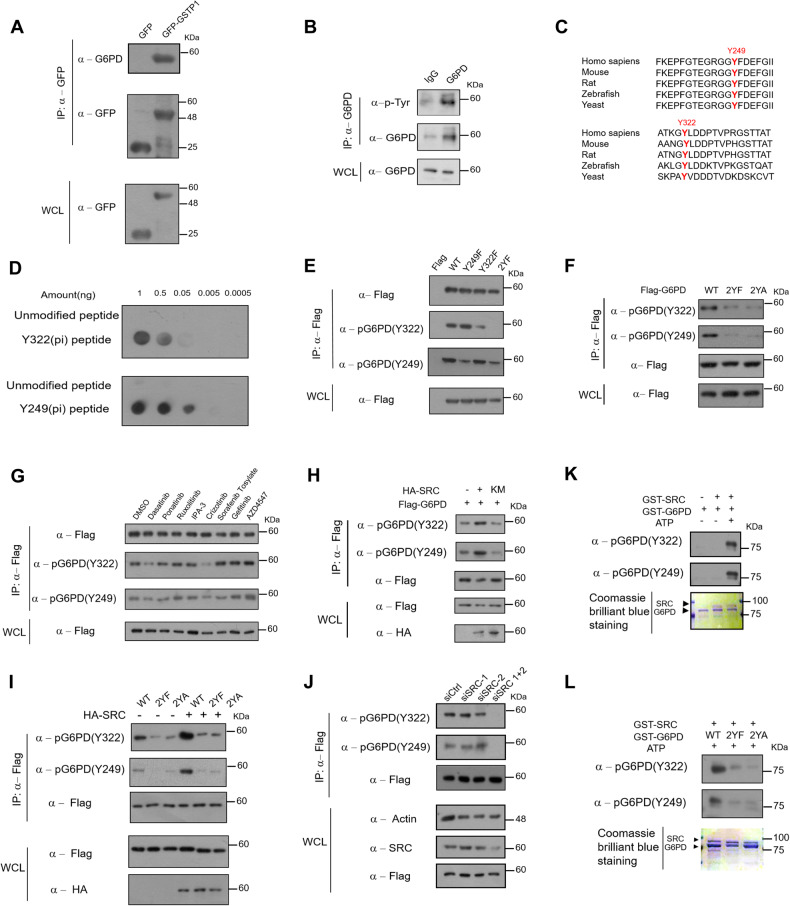
G6PD is dynamically Tyr-phosphorylated at Y249/Y322 by SRC via the GSTP1-G6PD-SRC axis. **A** Immuno-precipitates of GFP or GFP-GSTP1 analyzed by Western blots. **B** Endogenous G6PD protein is also Tyr phosphorylated. MCF-7 cells were seeded in 8 ten-cm dishes and cultured for two days. Cell lysates from 4 dishes were immunoprecipitated with control IgG, the rest with anti-G6PD antibodies. **C** Sequence alignment of phosphorylated Tyr residues among species. **D** Nitrocellulose membranes were spotted with indicated amounts of indicated peptides and probed with α-pG6PD Y249 and α-pG6PD Y322. **E** Flag-tagged WT and mutants were immunopurified with flag affinity (M2) beads and Western-blotted with α-pG6PD Y249 and α-pG6PD Y322 antibodies. **F** Tyr phosphorylation levels of G6PD WT vs. mutants were determined. **G** MCF-7 cells were treated with indicated Tyr kinase inhibitors for 12 h and Flag-tagged G6PD immunopurified to test phosphorylation levels. **H** HA-SRC (active) or HA-KM (kinase inactive SRC) were co-expressed with Flag-tagged G6PD in MCF-7 cells for 24 h, and Flag-tagged G6PD was immunopurified and immunoblotted with antibodies. **I** HA-SRC or HA-tag control empty plasmids were co-transfected with plasmids expressing WT (G6PD) and mutants (2YF/2YA) for 24 h, and Flag-tagged G6PD was immunopurified and immunoblotted with antibodies. **J** Two siRNA (#1 and #2) targeting SRC were transfected into MCF-7 cells. After 24 h, Flag-G6PD was also transfected for another 24 h. **K** Recombinant WT (G6PD) was expressed and purified from E. coil and incubated with or without recombinant SRC (active) and ATP. The mixture was subjected to analysis detecting phosphorylation of Y249 and Y322 (upper panel), and for Coomassie Brilliant Blue (CBB) staining that normalized input proteins (lower panel). **L** Recombinant WT (G6PD) and mutants (2YF/2YA) were incubated with recombinant SRC (active) and ATP. The mixture was subjected to analysis detecting phosphorylation at Y249 and Y322 (upper panel) and for CBB staining (lower panel) that normalized input proteins.

Mass spectrometry analyses of an immuno-enriched GFP-GSTP1 pull-down mixture revealed co-enriched G6PD (glucose-6-phosphate dehydrogenase) verified by immunoblotting (Fig. 2A and Fig. S2A). G6PD is the first rate-limiting enzyme of the PPP oxidative branch, and its enzymatic activity is intimately involved in regulating the PPP pathway that impacts cell proliferation [39, 40]. When over-expressed in MCF-7 cells cultured without or with glucose, Flag-tagged G6PD was phosphorylated mainly at Tyr residues and, to a lesser extent Ser/Thr residues, as revealed by anti-phospho-Tyr or -Ser/Thr antibodies that can immuno-enrich and detect phosphoproteins (Fig. S2B). Importantly, the same phosphorylation status was found in the endogenous G6PD (Fig. 2B). Mass spectrometry analyses of the immuno-enriched Flag-tagged-G6PD revealed Tyr phosphorylation at sites 249 and 322 (Y249/Y322) (Figure S2C). These sites are conserved from yeast to humans (Fig. 2C) and located in the C-terminus of the G6PD protein (Fig. S2D).

We generated specific polyclonal [anti-pG6PD(Y249) or anti-pG6PD(Y322)] antibodies against only phosphorylated epitopes, which was confirmed by dot-blot assays that exhibited no cross-reactivity with the unmodified epitopes (Fig. 2D). To investigate which phosphorylation site(s) played a major role, we mutated Y249/Y322 singly into Y249F or Y322 F (one residue to Phe), or doubly into 2YF or 2YA (both residues to Phe or Ala). The 2YF double mutant exhibited much reduced Tyr phosphorylation (Fig. 2E). Mutating a Tyr residue to Phe or Ala would completely eliminate phosphorylation (Fig. 2F), and NADP(H)-dependent dehydrogenases typically exist in dimer forms. Thus, residual Tyr phosphorylation signals associated with the 2YF mutant could be derived from an endogenous G6PD protein that formed a dimer with an ectopically expressed mutant, ditto similarly detected residual signals in the study. These results taken together suggest that Tyr 249 and 322 might be two major sites for phosphorylation, if not the sole.

To identify kinase(s) that modify G6PD, we screened a library of inhibitors for intracellular or receptor Tyr kinases such as Dasatinib [41] (SRC and ABL family), Ponatinib [42] (ABL family), Ruxolitinib [43] (JAK family), Crizotinib [44] (c-Met family) and AZD4547 [45] (FGFR family), and found that Dasatinib and/or Crizotinib detectably abrogated glucose-deprivation-triggered G6PD Y249/Y322 phosphorylation (Fig. 2G). Dasatinib is an inhibitor of SRC and ABL, but ABL is also inhibited by Ponatinib that did not inhibit the G6PD phosphorylation, so we considered the impacts of SRC and c-Met inhibited by Crizotinib excluding ABL. To further investigate the potential of SRC or c-Met to phosphorylate G6PD, HA-SRC or HA-c-Met was co-expressed with Flag-G6PD. It was found that HA-SRC, but much less so c-Met, phosphorylated G6PD at Y249/322 (Figure S2E). Over-expression of SRC, but not the kinase inactive mutant form, significantly induced G6PD phosphorylation (Fig. 2H). Also observed was much reduced Tyr phosphorylation with G6PD 2YF/2YA mutants as compared to the wild-type G6PD upon SRC over-expression in transfected cells (Fig. 2I). In addition, knockdown of the expression of the endogenous SRC in cells, albeit not to completion, much decreased the Tyr phosphorylation level of G6PD (Fig. 2J).

To determine whether SRC directly phosphorylates G6PD, we carried out an in vitro assay using GST-G6PD, GST-2YF, GST-2YA and GST-SRC expressed in and purified from E. coil. As shown, significant levels of Tyr phosphorylation of G6PD were only observed with the wild-type G6PD plus ATP (Fig. 2K), and the 2YF and 2YA mutants showed a drastic decline of G6PD phosphorylation by SRC through an in vitro assay (Fig. 2L). We conclude that the G6PD Y249/Y322 phosphorylation sites are the major Tyr sites and that SRC is most-likely the physiological intracellular kinase that phosphorylates the G6PD enzyme at Y249/Y322.

### A tripartite (GSTP1-G6PD-SRC) complex is modulated by lactic acid

**Fig. 3:**
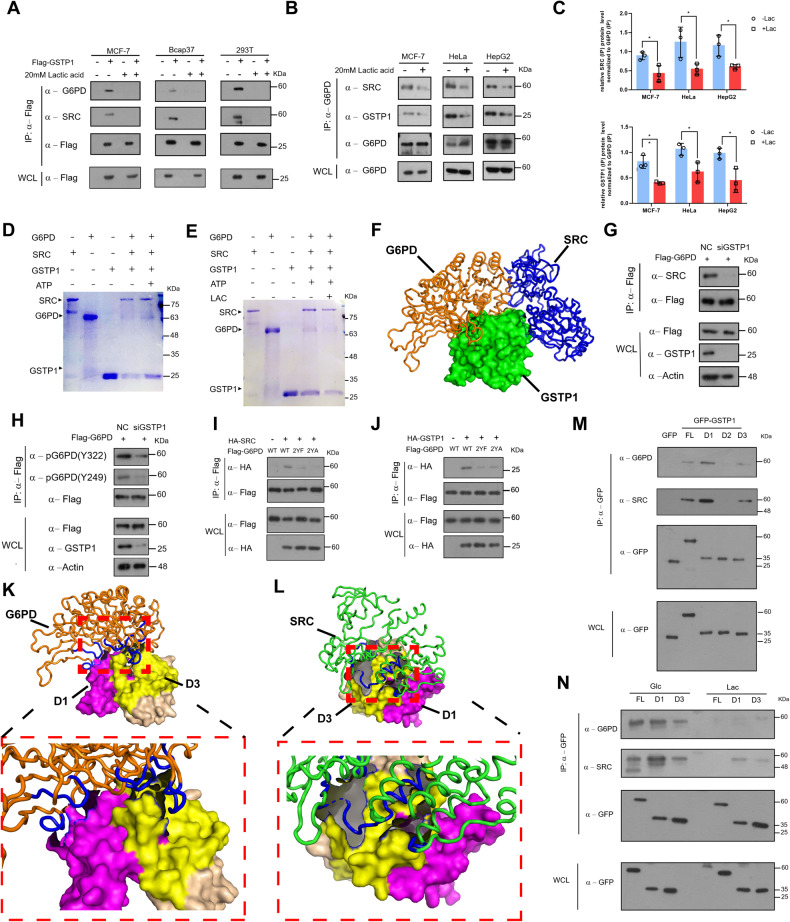
A tripartite (GSTP1-G6PD-SRC) complex formation is modulated by lactate. **A** Flag-tagged GSTP1 was overexpressed in cell lines (MCF-7, Bcap37, and 293 T) for 24 h. Cells were then incubated in glc medium (0.5 mM glucose) or lac medium (0.5 mM glucose with 20 mM lactic acid) for 12 h, and Flag-tagged GSTP1 protein immuno-purified for interaction detection. **B** MCF-7 cells/HeLa cells/HepG2 cells were incubated as in (**A**) for 12 h. Lysates were immuno-precipitated with anti-G6PD antibodies for endogenous interactions with GSTP1 and SRC. **C** Relative abundances of GSTP1 and SRC were obtained from 3 independent replicate experiments and normalized to immunoprecipitated G6PD endogenous protein in (**B**). ^*^p < 0.05, ^**^p < 0.01. **D** GST-SRC pull down to detect the GSTP1-G6PD-SRC interaction with or without ATP in vitro, as stained by CBB. **E** GST-SRC pull down to detect the GSTP1-G6PD-SRC interaction with or without lactic acid in vitro. **F** A modeled spatial arrangement of the GSTP1-SRC-G6PD complex. Green: GSTP1 (PDB ID: 3GUS); blue: SRC (PDB ID: 2H8H); Orange: G6PD(PDB ID: 2bhl). (G/H) Knockdown of GSTP1 decreases the interaction between G6PD and SRC (**G**) and overall G6PD phosphorylation level (**H**). **I**, **J** The interactions between GSTP1 (SRC) and G6PD decreased in G6PD mutants (2YF/2YA) in cells. HA-SRC (**I**), HA-GSTP1 (**J**), or HA-tagged empty control plasmids were co-transfected with WT (G6PD) or mutants (2YF/2YA) constructs for 24 h, and Flag-tagged G6PD was immunopurified and immunoblotted with indicated antibodies. **K**, **L** Modeled spatial arrangement of the GSTP1-SRC (**L**) or GSTP1-G6PD (**K**) complex. Orange: G6PD; Green: SRC; Pink: the 1-70AA region of GSTP1; Yellow: the 141-210AA region of GSTP1. **M** GFP, GFP-fused full length or indicated domains of GSTP1 were overexpressed in MCF-7 cells and immunoprecipitated by anti-GFP beads and examined through Western blots to check the interaction among G6PD, GSTP1 and SRC. **N** Proteins as indicated in (**M**) were overexpressed in MCF-7 cells, and cells treated with glucose-deprived (0.5 mM) media without or with 20 mM lactic acid to check the dynamic change of the tri-complex formation through Western Blots.

We ectopically expressed Flag-tagged GSTP1 in three cell lines (MCF-7, Bcap37, and 293 T) and used cell lysates mixed with M2 beads to immuno-precipitate (IP) tagged GSTP1 with or without lactic acidosis. We found a tripartite complex among GSTP1, G6PD and SRC, which was especially prevalent when cells were glucose-deprived, and lactic acid attenuated the interaction (Fig. 3A). Then we used three cell lines (MCF-7, HeLa, and HepG2) to independently detect lactic acid-attenuated tripartite interactions of endogenous proteins (Fig. 3B, C, Fig. S3A). Bacterially expressed and purified GST-SRC, His-G6PD and His-GSTP1 were employed to investigate whether the in vivo interactions can be recapitulated in vitro, and we showed that SRC, G6PD and GSTP1 indeed formed a tripartite complex (Fig. 3D), which was impeded by lactic acid (Fig. 3E) suggesting that the tripartite complex is negatively regulated by lactic acid.

Computer modeling of the tripartite complex suggested that GSTP1 bridged the interaction between G6PD and SRC (Fig. 3F). Indeed, knockdown of GSTP1 expression reduced the interaction between G6PD and SRC (Fig. 3G) and decreased phosphorylation of G6PD (Fig. 3H), and the G6PD (2YF/2YA) mutants showed much-weakened interactions with SRC or GSTP1 (Fig. 3I, J). In addition, computer modeling of the tripartite complex demonstrated that G6PD (PDB ID: 2bhl) or SRC (PDB ID: 2H8H) interacted with the D1 and D3 regions of GSTP1 (Fig. 3K, L). These regions are also domains predicted and confirmed to contain binding pockets for lactate (Fig. 1L).

The lactate-binding nature of GSTP1 and a tripartite (GSTP1-G6PD-SRC) complex suggest that lactate functionally modulates the assembly of the complex. Co-immunoprecipitation analyses validated that the D1 and D3, but not the D2, regions of GSTP1 interacted with G6PD and SRC (Fig. 3M). Moreover, these interactions were subject to attenuation by lactic acid (Fig. 3N). Therefore, most-likely lactate can non-covalently trigger a GSTP1 conformation changes that could negatively affect the formation of the tripartite GSTP1/G6PD/SRC complex.

### Lactic acid sustains redox homeostasis via a Lac-GSTP1-G6PD axis

**Fig. 4:**
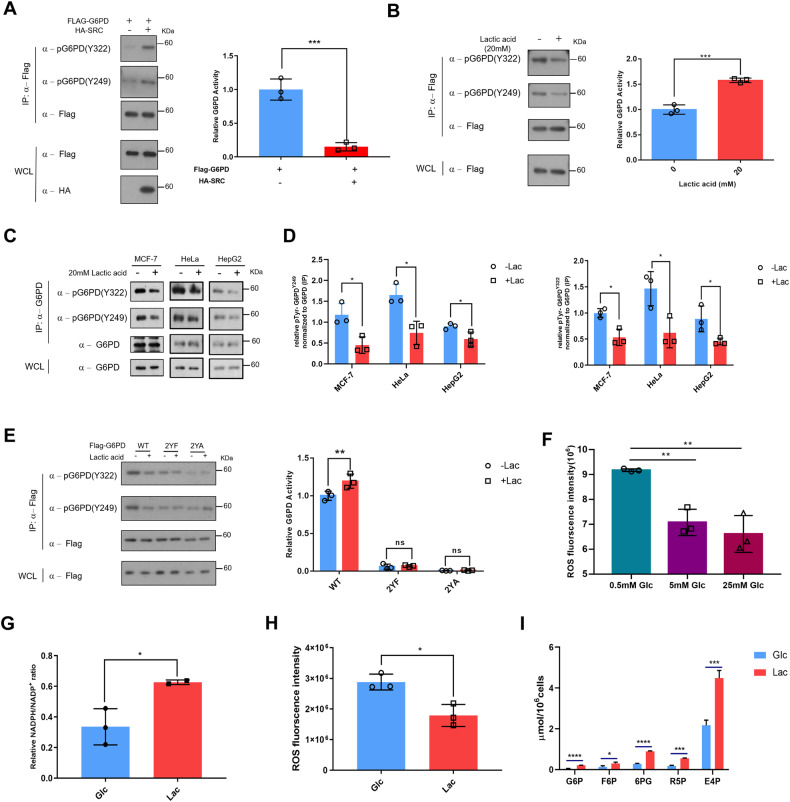
Lactic acid modulates the catalytic properties of G6PD and switches cells to a reprogrammed PPP mode through GSTP1. **A** G6PD activity upon SRC over-expression as measured by catalytic activity of immuno-purified/Flag peptide-eluted Flag-tagged G6PD (Left panel). Results are mean ± SD of triplicate experiments. The p value was analyzed by Student’s t test. ***p = 0.0009. Right panel showed that the phosphorylation of G6PD under SRC over-expression. **B** MCF-7 cells were transfected with Flag-G6PD for 24 h and switched to glucose-deprived media with or without 20 mM lactic acid for another 12 h. Flag-G6PD was immunopurified, the phosphorylation status (Right panel) and the activity (Left panel) were determined. Relative enzymatic activity was presented as mean ± SD of triplicate independent experiments and analyzed by unpaired Students’ test. *p = 0.0138. **C**, **D** MCF-7 cells/HeLa cells/HepG2 cells were treated with or without 20 mM lactic acid. The lysates were immunoprecipitated with G6PD antibody (**C**). The phosphorylation level was determined through normalization with total G6PD (**D**). Two other independent experiments are shown in supplementary materials. ^*^p < 0.05. **E** Lactic acid reduces Y249 and Y322 phosphorylation of WT but not its mutants in MCF-7 cells (Left panel), and the activities of 2YF and 2YA versus the WT were determined (Right panel). Two-way ANOVA was applied to analyse the results. **p_(WT -lac VS WT +lac)_ = 0.0038. **F** The ROS levels were determined under different concentrations of glucose. **p < 0.01. **G** Lactic acid increases the NADPH/NADP^+^ ratio. The NADPH/NADP^+^ ratios were determined at OD 570 nm. Data are presented as mean ± SD of triplicate experiments and analyzed by unpaired Students’ test. *p = 0.0459. **H** Lactic acid reduces the ROS level. Data are presented as mean ± SD of triplicate experiments and analyzed by unpaired Students’ test. *p = 0.0131. **I** Abundances of key PPP metabolites were determined, *n* = 3. Results are mean ± SD of triplicate experiments. The *p*-value was analyzed by Student’s *t*-test.

To establish a correlation between G6PD enzymatic activity and phosphorylation level, we ectopically expressed HA-SRC along with Flag-G6PD in breast cancer cells. HA-SRC expression decreased the G6PD catalytic activity presumably via increasing its phosphorylation level under glucose deprivation (Fig. 4A). To further explore a modulation function of lactic acid on PPP, we investigated its effect on the G6PD enzymatic activity. To this end, we treated the Flag-G6PD over-expressing cells with 20 mM lactic acid and immuno-purified the protein. Elevating lactic acid levels reduced G6PD Tyr phosphorylation and augmented the G6PD enzymatic activity (Fig. 4B) via increasing the affinity for its substrate glucose-6-phosphate (G6P) (Fig. S4A). Additionally, the phosphorylation of the endogenous G6PD was significantly reduced by increasing the lactic acid levels in 3 independently tested (cervical HeLa, liver hepatocellular carcinoma cell HepG2 and breast cancer MCF-7) cell lines (Fig. 4C, D and Fig. S4B). Moreover, the phosphorylation status of the 2YF/2YA mutants largely remained unchanged in MCF-7 cells (Fig. 4E). Collectively, these results suggest that G6PD is subject to dynamic phosphorylation at two tyrosine residues (Y249 and Y322) under lactic acidosis.

However, the G6PD 2YF mutant exhibited a ~ 90% reduction in catalytic activity (Fig. S4C) in conjunction with a ~ 1.5-fold increase in Km for the substrate (Fig. S4D). In silico modeling reveals that the two relevant Tyr residues are spatially close to the substrate binding pocket of G6PD (Fig. S4E). The Missense Software dictates that substitution of the two residues (Y to F) would create a contraction of a catalytic cavity volume by 113.616 Å^^^3 and disrupt all potential side-chain/side-chain H-bonds with the two buried Tyr residues. Hence, the 2YF mutant might impede G6P going into the catalytic cavity but decreasing its phosphorylation status. We nevertheless included the 2YF/2YA mutants in functional assays of G6PD, not to demonstrate a role as a phosphorylation-defective surrogates but to show that G6PD catalytic activity is essential for the Lac-GSTP1-G6PD axis to regulate the pentose phosphate pathway.

When glucose was scarce, the levels of ROS were elevated (Fig. 4F), which could be eliminated by NADPH and damage to the growth of breast cancer cells. Moreover, cells with glucose deprivation cultured with lactic acid exhibited a higher NADPH/NADP^+^ ratio (Fig. 4G) and decreased ROS levels (Fig. 4H) as compared to control cells with scarce glucose only. Metabolomics analyses showed that the metabolites such as 6PG, R5P and E4P of the PPP pathway were raised under lactic acidosis (Fig. 4I), supporting a notion that lactic acid regulates redox homeostasis via PPP. Previous results indicated that GSTP1 was a lactate sensor (Fig. 1), and a GSTP1-mediated lactic acid signaling regulated the tri-complex and phosphorylation of G6PD (Fig. 2 and Fig. 3), further suggesting that lactic acid signals to GSTP1 to regulate the redox homeostasis via activating the pentose phosphate pathway.

### A GSTP1-mediated lactic acid signaling promotes breast cancer cells proliferation

**Fig. 5:**
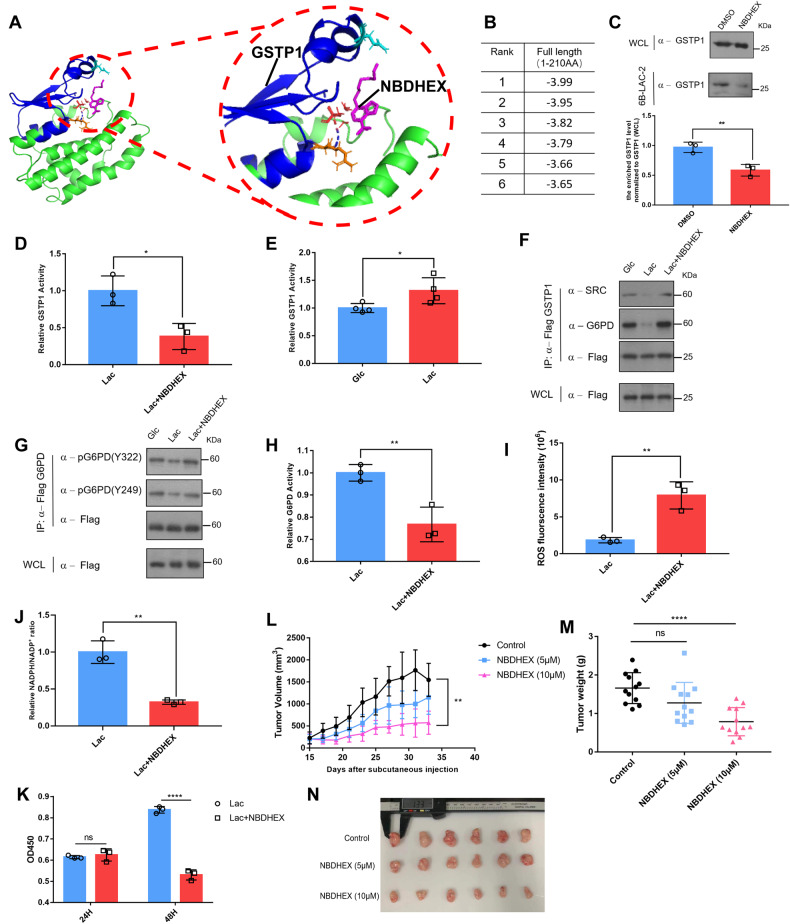
A GSTP1-mediated lactic acid signaling promotes breast cancer cells proliferation. **A** Modeled structure of parts of GSTP1 (PDB ID: 3GUS) with NBDHEX labelled in pink, the blue area represents parts of GSTP1 involved in NBDHEX binding. **B** Top 6 ranked binding energy values (kcal/mol) are shown. **C** GSTP1 treated with or without inhibitor NBDHEX was enriched via 6B-LAC-2 beads and examined through Western blots (Upper panel). The enriched GSTP1 via 6B-LAC-2 beads were determined (Lower panel). Data are presented as mean ± SD of triplicate experiments and analyzed by unpaired Students’ test. **p = 0.007. **D** The enzymatic activity of GSTP1 was examined with or without NBDHEX. *p = 0.0162. **E** The enzymatic activity of GSTP1 was examined with or without lactic acid in glucose-deprived cells. *p = 0.0446. **F** Flag-tagged GSTP1 was overexpressed in MCF-7 cell lines for 24 h. Cells were then incubated in glc medium (0.5 mM glucose) or lac medium (0.5 mM glucose with 20 mM lactic acid) or NBDHEX medium (0.5 mM glucose, 20 mM lactic acid and 10 µM NBDHEX) for 12 h, and Flag-tagged GSTP1 protein immuno-purified for interaction assays. **G** Flag-tagged G6PD was overexpressed in MCF-7 cell lines for 24 h. Cells were then incubated in glc medium (0.5 mM glucose) or lac medium (0.5 mM glucose with 20 mM lactic acid) or NBDHEX medium (0.5 mM glucose, 20 mM lactic acid and 10 µM NBDHEX) for 12 h, and the phosphorylation status was determined. **H** MCF-7 cells overexpressing flag-tagged G6PD were pretreated with or without 10 µM NBDHEX for 1 h, switched to 20 mM lactic acid and 10 µM NBDHEX for 12 h and enzymatic activity of G6PD measured. Data are presented as mean ± SD of triplicate experiments and analyzed by unpaired Students’ test. **p = 0.0096. **I**, **J** After treatments as in (**D**), the ROS levels (**I**) and NADPH/NADP^+^ ratio (**J**) were measured and analyzed by unpaired Students’ test. **p_(ROS)_ = 0.0049; **p_(NADPH/NADP+ ratio)_ = 0.0017. **K** MCF-7 cells were seeded in the 96-well plate, treated with or without 10 µM NBDHEX in media containing 20 mM lactic acid for indicated days, and measured at OD 450 nm by using CCK-8 kit. Data are presented as mean ± SD of triplicate experiments and analyzed by multiple t test. ****p (48H Lac vs 48H Lac NBDHEX) = 0.000049. **L** Tumor sizes were measured from day 15. Data are presented as mean ± SD of triplicate experiments and analyzed by unpaired Students’ test. **p_(Control vs 10µM NBDHEX)_ = 0.0021. **M** Tumors were weighted at day 34. Data are presented as mean ± SD of triplicate experiments and analyzed by unpaired Students’ test. ****p_(control vs 10µM NBDHEX)_ < 0.0001. **N** Pictures illustrating breast tumor burdens in nude mice grafted with MCF-7 cells and subjected to no or NBDHEX treatment.

GSTP1 is an enzyme intimately involved in resistance to anti-cancer drugs [28, 46], and its inhibitor NBDHEX can suppress the catalytic activity and prevent it from interacting with signaling molecule(s) [47, 48]. Computer modeling showed that NBDHEX interacted with the 1-70AA region of GSTP1 (Fig. 5A), which was the similar with previous paper [49] and overlaps with a verified binding region between lactate and GSTP1 but involving different amino acids. The standard change of Gibbs free energy was higher than that with the lactate group (Fig. 5B). Thus, we presumed that NBDHEX would prevent lactate from interacting with GSTP1. Indeed, NBDHEX inhibited the interaction between lactate and GSTP1 (Fig. 5C and Fig. S5A) and the GSTP1 catalytic activity (Fig. 5D) and, conversely, lactic acid promoted the activity of GSTP1 (Fig. 5E), suggesting that NBDHEX impedes a GSTP1-mediated lactic acid signaling pathway. To verify the inhibitory effects of NBDHEX on lactic acid signaling, we found that NBDHEX maintained the interaction of the tri-complex (Fig. 5F) and the phosphorylation of G6PD (Fig. 5G) even under lactic acidosis. Thus, NBDHEX significantly decreased the G6PD catalytic activity (Fig. 5H) known to be augmented by lactic acid. In addition, the ROS levels were elevated (Fig. 5I) along with a decreased NADPH/NADP^+^ ratio (Fig. 5J). NBDHEX also suppressed cell proliferation in glucose-deprived media supplemented with lactic acid (Fig. 5K). In nude mice grafted with MCF-7 cancer cells, growth of tumors in mice administered with 10 µM NBDHEX was significantly impeded (Fig. 5L–N). These results taken together strongly support a hypothesis that a GSTP1-mediated lactic acid signaling pathway is involved in promoting the G6PD catalytic activity and PPP pathway under lactic acidosis.

Summarily, the above results support that, when glucose is deprived and without a tumor metabolic micro-environment lactic acidosis, GSTP1 forms a tripartite complex with G6PD and SRC (Fig. 3) leading to G6PD Tyr-phosphorylation (Fig. 2) with a reduced catalytic activity thus the reducing power NADPH, and much narrower PPP pathway (Fig. 4). Lactate binds with GSTP1 (Fig. 1) and attenuates the tripartite complex (Fig. 3) and G6PD phosphorylation (Fig. 4), which would free up the G6PD enzyme and boost its catalytic activity to augment the PPP pathway. In addition, a Lac-GSTP1-G6PD axis sustains a redox homeostasis in cancer cells, which is beneficial to tumor growth (Graphical Abstracts). This explains how lactic acid is capable of robustly promoting proliferation when cancer cells encounter severe glucose deprivation and, probably, other metabolic stresses.

### The G6PD phosphorylation is positively correlated with G6PD-SRC-GSTP1 complex in ER/PR positive type or HER negative types of breast cancer

**Fig. 6:**
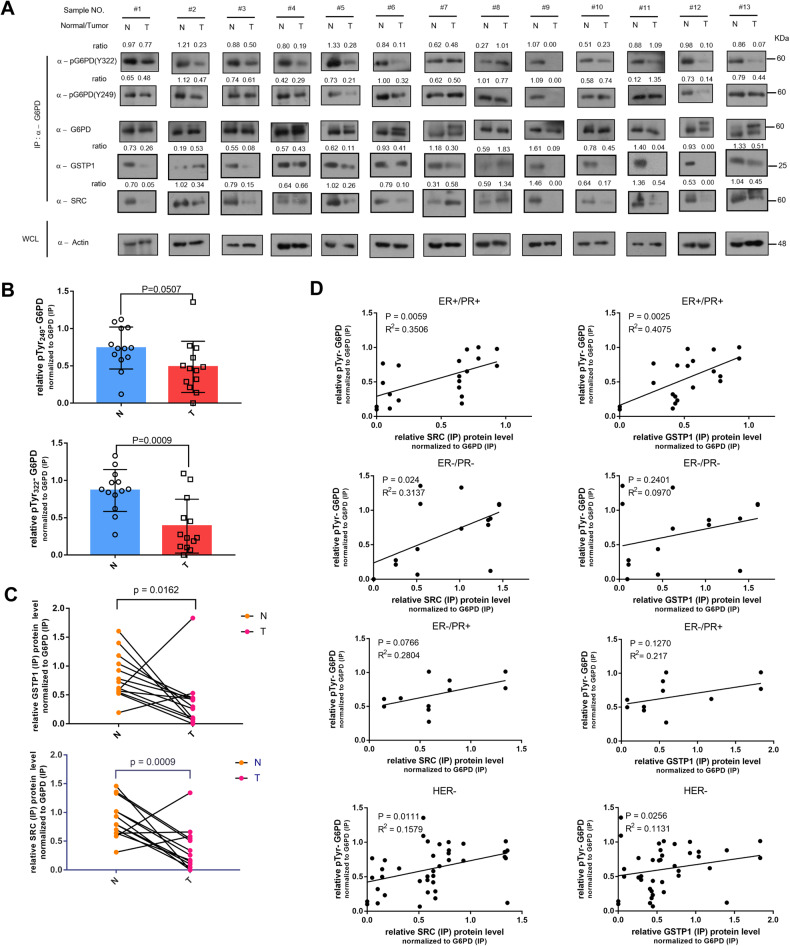
The phosphorylation status of G6PD was positively related to the immunoprecipitated SRC/GSTP1 in the ER/PR positive type or Her (-) type of breast cancer. **A** Immunoprecipitated SRC/GSTP1 and Y249/Y322 phosphorylation status of G6PD that was immuno-enriched from clinical human breast cancer samples, as determined by Western-blots against the tumor (T) and adjacent normal tissues (N). **B** The G6PD Tyr phosphorylation status at 2 sites in (**A**) were quantified and statistically analyzed. Data are normalized to G6PD (IP) protein, presented as mean ± SD and analyzed by unpaired Students’ test, *n* = 13 pairs. *P*_(Y249)_ = 0.0507, *P*_(Y322)_ = 0.0009. **C** The immunoprecipitated GSTP1 and SRC expression levels in (**A**) were quantified and statistically analyzed. Data are normalized to G6PD (IP) protein, presented as mean ± SD and analyzed by unpaired Students’ test, *n* = 13 pairs. *P*_(SRC)_ = 0.0009, P_(GSTP1)_ = 0.0162. **D** A possible correlation between relative SRC/GSTP1 (IP) levels and relative p-Tyr levels of G6PD was determined in various types of breast cancer samples. Data are analyzed and shown by Pearson Correlation Coefficient. ER + /PR + : 5pairs; ER-/PR-: 4 pairs; ER-PR + : 3 pairs; HER-: 10 pairs.

Molecular sub-typing of breast cancer heavily relies on the expression patterns of estrogen receptor (ER), progesterone receptor (PR) and human epidermal growth factor receptor 2 (HER) [50]. To investigate a clinical relevance of the lactic acid-responsive G6PD^Y249/322^ Tyr phosphorylation and G6PD-SRC-GSTP1 complex, and to establish their potential correlation with breast cancer types, we obtained 13 human breast cancer (T) samples along with adjacent tissues (N) characterized to be normal, and made lysate for detailed IP analyses (Fig. S6A). The G6PD Y249/Y322 phosphorylation was drastically decreased or even absent in most tested human breast cancer samples (Fig. 6A, B). Moreover, the levels of immuno-enriched SRC and GSTP1 were lower in human breast cancer samples than in normal tissues when normalized to endogenous G6PD protein controls (Fig. 6A,C), which is consistent with in vitro data implying that the dynamic G6PD Tyr phosphorylation was related to a changing tripartite complex in response to lactic acid.

We next examined the relationship between the IP-enriched SRC/GSTP1 protein and the overall G6PD^Y249/322^ phosphorylation levels in these clinical samples upon classifying them into types based on ER/PR/HER2 expression patterns. The progesterone receptor is the downstream target of the estrogen receptor, so we take progesterone receptor and estrogen receptor together into consideration. SRC/GSTP1 expression and G6PD^Y249/322^ phosphorylation were positively correlated in breast cancer tissues characterized to be ER (+) and PR (+) or HER (-); however, this correlation was not evident in ER(-)/PR(-) or ER(-)/PR(+) or HER (+) tumor tissues (Fig. 6D, Fig. S6B). Further, the IP-enriched SRC/GSTP1 protein and G6PD^Y249/322^ Tyr phosphorylation status were strongly and positively correlated with the breast cancer that exhibit ER expression and PR expression, to which our experimental cell line MCF-7 belongs. Hence, the G6PD^Y249/322^ Tyr phosphorylation sites and LAC-G6PD-SRC-GSTP1 axis are potentially a novel target for the ER/PR positive type or Her (-) type of breast cancer.

## Discussion

Many a signaling pathway necessitates multiplex protein-protein associations via dynamic complex that receive and forward diverse proliferative or other signals, which in essence function as “signalosomes”. We, for the first time, show that the tumor metabolic microenvironment lactic acid activates a “signalosome” comprising GSTP1, SRC and G6PD by attenuating the complex, which facilitates a reprogrammed PPP to promote cancer cell growth. Such a dynamic pattern of a complex is also manifested in certain breast cancer subtypes of human clinical specimens. Hence, these findings unravel crucial roles of lactic acid during malignancies and imply strategies for cancer intervention through metabolic means by targeting individual components involved in, or by perturbing the formation of, the complex as a druggable “signalosome”.

### Roles of lactic acid during malignancies

Wealth of evidence points to essential roles of lactic acid during malignancies. Aside from serving a fuel molecule [51] or a carbon source for other metabolites, and aside from an involvement in gene regulation transcriptionally or post-translationally [52], lactic acid can function without net chemical consumption thus in essence a signaling molecule. Indeed, we found that the proton reduced the glycolysis flux by suppressing the catalytic functions of multiple glycolytic enzymes, whereas a high level of lactate facilitated the glycolysis towards reaching an equilibrium [19]. Such a combinatorial form of metabolic regulation employing both elements of lactic acidosis would in theory limit a glycolytic flux preventing further glucose fermentation in the direction of lactate production thus intercepting glycolytic intermediates. Given ample glucose (a likely scenario with outer-sphere cells of solid tumors), cells can afford the Warburg Effect to generate sufficient intermediates for biomass building; however, the glycolytic flux must be kept in check in case of glucose deprivation (a scenario with inner-sphere cells of solid tumors). In the latter scenario, the lactic acidosis-based interception mechanism would allow more glycolytic intermediates to be diverted to biomass building, which, as our current work unravels, also includes metabolizing G6P towards the PPP for nucleotides synthesis (R5P) and reducing power NADPH (anti-oxidation and reductive biosynthesis). Thus, lactic acid constitutes a crucial part of a signaling cascade that allows optimal partitioning of glucose metabolism intermediates for energetics and reductive biosynthetic and anti-oxidation capabilities, and in so doing plays essential roles during malignancy of solid tumors.

This work mechanistically addresses the question regarding how the lactic acid signaling functions. G6PD, the first rate-limiting enzyme of the PPP oxidative branch, is regulated by phosphorylation at Y249/322 by SRC (Fig. 2). GSTP1 up-regulates the G6PD phosphorylation by recruiting SRC as part of the tripartite complex, which might completely impede PPP; however, lactate binds to GSTP1 (Fig. 1) that in turn attenuates the tripartite GSTP1-SRC-G6PD interaction (Fig. 3), altering the G6PD phosphorylation status and augmenting NADPH levels by up-regulating the G6PD catalytic activity via GSTP1 (Figs. 4, 5). We emphasize that both elements of lactic acidosis contribute: a high level of lactate facilitates an equilibrium of glycolysis and proton squelches a number of glycolytic enzymes, which in a combinatorial way intercepts more intermediates for biosynthesis especially diverting G6P to PPP for more R5P and NADPH. Hence, lactic acid can function as a tumor metabolic micro-environment that reverses the above-mentioned PPP impediment, which in turn robustly supports proliferation of (severely) glucose-deprived cells, a situation that inner-sphere solid tumor cells quite often encounter.

### Function of the GSTP1-G6PD-SRC complex as a metabolic “signalosome”

Our work implies that GSTP1 plays a dominant role linking G6PD and SRC to form a “signalosome” that provides a basis, under metabolic stresses, for robust cancer cell proliferation upon lactic acid signaling, which could regulate many aspects of cellular metabolism. Given roles of lactate in promoting redox (re)balance and PPP pathway via GSTP1 (Fig. 5), the defined GSTP1 regions involved in binding with lactate (Fig. 1) are likely potential targets for blocking the lactic acid signaling, paving the way of searching for antagonist(s). Of note, defined GSTP1 lactate binding regions are also regions that mediate interactions with G6PD/SRC.

#### GSTP1

Of the trio involved in the above-mentioned “signalosome”, high GSTP1 expression levels have in fact been reported in diverse human malignancies including breast, colon and ovarian cancer, and an involvement of GSTP1 in tumorigenesis and drug resistance in general has long been recognized [53, 54]. In other studies, NBDHEX allosterically binds to GSTP1 leading to activation of JNK via the interaction of the JNK-GSTP1 axis, and the conformation of GSTP1 is modulated upon binding to certain anti-cancer drugs [55]. But the interaction between GSTP1 and JNK didn’t change when treated with or without lactic acid (Fig. S7A). In addition, GSTP1 can mediate the S-glutathionylation of some proteins and interact with them [56] such as Keap1 [57] and PKM2 [58]. We also checked the S-glutathionylation of G6PD and SRC under lactic acidosis. G6PD could be S-glutathionylated (Fig. S7B) and the modification level decreased under lactic acid (Fig. S7C). Previous paper reported that GSTP1 promoted the S-glutathionylation of SRC under TNF-α stimulation [59], but is not found under lactic acidosis in our paper (Fig. S7D). Hence, especially given a crucial role of GSTP1 in receiving and forwarding the lactic acid signaling as we unravel, intervention targeting the GSTP1 protein could impede multiple metabolic pathways in diverse cancers. Furthermore, small molecules such as LAS17 [60] that suppresses the GSTP1 enzymatic activity or protein expression are predicted to have therapeutic potentials. We observed a tumor suppressing activity by a GSTP1 enzyme inhibitor NBDHEX in animal models, presumably by blocking the function of GSTP1 as a lactic acid sensor that impacts the GSTP1-G6PD-SRC complex (Fig. 5).

#### SRC

The oncoprotein c-SRC once activated is capable of potently promoting cell proliferation, adhesion and migration [61, 62]. SRC is known to take part in regulating the activities of glycolytic enzymes thus overall flux. In scenarios with activated c-SRC, however, the ROS levels are dramatically elevated [63, 64]. A balanced ROS is vital for cellular physiology and is regulated via redox pairs, particularly by fluctuating the NADPH/NADP^+^ ratio and/or NADPH/NADP^+^ recycling in conjunction with the GSH/GSSH recycling [65, 66]. In extreme, cancer cells acquire anti-ROS capacities by establishing new redox balances that can counteract even more than 100-fold-elevated ROS levels. This might be how augmented PPP comes into play: generating more NADPH or facilitating an NADPH/NADP^+^ recycling while accumulating the building block R5P for DNA/RNA synthesis. It is especially crucial if cancer cells encounter scarce glucose but necessitates a signaling from lactic acid to facilitate the PPP. SRC inhibited the G6PD catalytic activity via Tyr-phosphorylation (Fig. 4A). Manifesting opposing effect(s) of a presumed onco-protein as it may seem, it allows lactic acid to be sensed by GSTP1 to activate a cascade leading to augmented PPP that overcomes ROS levels elevated by activated c-SRC. That lactic acid attenuated the G6PD Y249/Y322 phosphorylation and boosted its enzyme activity (Fig. 4B) is consistent with a scenario in which the elevated ROS levels by c-SRC activation are squelched by augmented PPP under glucose deprivation and with a lactic acid signaling.

#### G6PD

G6PD is a rate-limiting enzyme of the PPP, and its expression is elevated in diverse cancers. The TCGA database positively correlated elevated abundance of G6PD with poor prognosis of cancer patients [67, 68]. We identified two new (Y249 and Y322) phosphorylation sites (Fig. 2) influenced by the tumor metabolic microenvironment lactic acid sensing by GSTP1 (Fig. 1), which frees up G6PD from a tripartite complex (Fig. 3) to facilitate the PPP oxidative branch (Fig. 4). Thus, a GSTP1-G6PD-SRC complex might allow this metabolic “signalosome” to orchestrate responses of cancer cells to diverse forms of metabolic stresses typified by glucose deprivation and, with a lactic acid-triggered mechanism, facilitate biomass building and redox (re)balance to support robust cell proliferation (Graphical Abstracts). Our newly-defined G6PD Y249/Y322 phosphorylation sites and a regulatory mechanism centered upon the GSTP1-G6PD-SRC complex, especially given observations on clinical samples, could be more physiologically relevant during malignancy. However, G6PD reportedly undergoes modifications such as phosphorylation at Y112 and Y428 [69, 70] in HCT116 cells, acetylation at K403 [71] in leukaemia cells and glycosylation at S84 in lung cancer cells [72]. The above modifications reported were detected under different growth factors stimulation, and might not reflect the genuine situation of cancer cells that quite often encounter metabolic stresses in diverse forms. Therefore, Such difference may be caused by different cell types or other factors, which needs to be investigated later.

### Perspectives

A more detailed description of molecular impacts exerted by GSTP1 as a lactic acid sensor remains to be elucidated, along with the normal physiological function of the LAC-G6PD-GSTP1 axis. Our findings imply that GSTP1 plays key roles in regulating metabolism subject to modulation upon sensing the tumor metabolic microenvironment lactic acidosis. In a hindsight, all components of the tripartite complex are metabolic enzymes that participate in maintaining the redox balance. Targeting cellular redox systems, in particular those that involve the NADPH/NADP^+^ pair, might pave the way for more efficacious metabolic intervention than targeting cellular energetics. Our findings would broaden strategic options for tumor intervention by metabolic means.

## Methods and Materials

### Human breast cancer samples

Human breast cancer samples were obtained from the Breast Surgery Department of the Second Affiliated Hospital of Zhejiang University School of Medicine with approval of the ethics committee of the hospital and informed consents from all patients. In total, 13 cases of human breast cancer tissues were obtained, all from women, and all individuals were over 40 years old. Each breast cancer sample was paired with an adjacent normal sample from the same patient. Prior to analyses, all samples were stored in liquid nitrogen to keep the integrity of cellular proteins.

### Cell lines

Human breast cancer cell lines MCF-7 and Bcap37 were gifts from, respectively, Laboratories of DONG Chenfang and HU Xun of Zhejiang University. Other cell lines (HEK293T, HepG2 and HeLa) were purchased from Cell Bank of Type Culture Collection of the Chinese Academy of Sciences, Shanghai, China. All cell lines were proven to be free of mycoplasma contamination.

### Cell culture conditions

Unless otherwise noted, cells were cultured in Dulbecco Minimal Essential Medium (DMEM) supplemented with 1% penicillin/streptomycin sulfate and 10% fetal bovine serum (FBS) in a 37 °C incubator with 5% CO_2_ atmosphere. For plasmid and siRNA transfection assays, media were devoid of antibiotics. For glucose starvation, media with normal glucose level were removed, cells washed twice with 1X PBS, and glucose-deprived media (final glucose level at 0.5 mM) with or without lactic acid added for 12 h. For kinase inhibitor treatments, cells were administered by the following: Dasatinib, 5 µM, 12 h, Selleck Chemicals; Ponasatinib, 5 µM, 12 h, Selleck Chemicals; Ruxolitinib 5 µM,12 h, Selleck Chemicals; IPA-3, 10 µM, 12 h, Selleck Chemicals; Crizatinib, 10 µM,12 h, Selleck Chemicals; Sorafenib Tosylate, 20 µM,12 h, Selleck Chemicals; Getifinib, 2 µM,12 h, Selleck Chemicals; AZD4547, 20 µM,12 h, Selleck Chemicals,NBHDEX, 5 µM or 10 µM, 12 h, MCE. All inhibitors were used at dosages suggested by the suppliers.

### Antibodies

The following antibodies were purchased from Cell Signaling Technology: GSTP1 (3F2) (3369; 1: 2,000), SRC (36D10) (2109; 1:2,000), Rabbit IgG (Light-Chain Specific) (D4W3E) (93702; 1:2,000), Mouse IgG (Light-Chain Specific) (D3V2A) (58802;1:2,000), and (Ser/Thr) Phe (9631;1:1,000). The following antibodies were purchased from Santa Cruz Biotechnology: p-Tyr (PY99) (sc-7020; 1:1,000), and HA-tag (F-7) (sc-7392; 1:5,000). G6PD (SAB5300459; 1:8,000) was purchased from Sigma-Aldrich; another GSTP1 (ab153949; 1:2,000) was purchased from Abcam; Flag (30502ES20; 1:10,000) was obtained from YEASEN; GFP (R1312-2; 1:4,000) and Actin (EM21002; 1:10,000) was purchased from HuaAn Biotechnology. To create site-specific antibodies for Tyr 249 and 322 of G6PD (Anti-pG6PD (Y249) and Anti-pG6PD (Y322)), synthesized peptides CEPFGTEGRGGY(pi)FDEF and CGEATKGY(pi)LDDPTVP (HuaAn Biotechnology) were coupled to a protein carrier prior to immunization (2 rabbits/antigen). High titer anti-sera that specifically recognized the pTyr-containing epitopes were obtained after one initial immunization and three boosts, and were stored at −70 °C.

### Plasmid constructs

Plasmid encoding *SRC* was purchased from Sino Biological, and SRC mutant (KM) was kindly provided by Dr. ZHAO Bin (Zhejiang University). Human full-length *G6PD* cDNA, its mutants, full-length *GSTP1* and its truncated GSTP1 versions were cloned into the PXJ40-based Flag-tag, HA-tag and GFP-tag vectors for cell transfection; pGEX-4T1 vector was used for GST-G6PD proteins (WT, 2YF or 2YA) expression in bacteria. GST tagged SRC protein was purchased from Sino Biological. His tagged GSTP1 and His tagged G6PD protein was also obtained from MCE.

The primers (forward and reverse) for constructing plasmids are listed below:

human *G6PD* (5’-ATATAAGCTTATGGCAGAGCAGGTGGCCCTGA-3’ and 5’-ATATATACCCGGGTCAGAGCTTGTGGGGGTTCACCC-3’);

human *G6PD* Y249F (5’-GGGCTTTTTCGATGAATTTGGGATCATCCGGG-3’ and 5’-ATTCATCGAAAAAGCCCCCGCGACCCTCAGTG-3’);

human *G6PD* Y322F (5’- AAAGGGTTCCTGGACGACCCCACGGTGCCCCG

-3’ and 5’- TCGTCCAGAGGCCCTTTGGTGGCCTCGCCCTC -3’);

human *G6PD* Y249A (5’-GGGCGCTTTCGATGAATTTGGGATCATCCGGG-3’ and 5’-ATTCATCGAAAGCGCCCCCGCGACCCTCAGTG-3’);

human *G6PD* Y322A (5’-AAAGGGGCCCTGGACGACCCCACGGTGCCCCG-3’ and 5’- TCGTCCAGGGCCCCTTTGGTGGCCTCGCCCTC -3’);

human *GST-G6PD* (5’-ATATGAATTCATGGCAGAGCAGGTGGCCCTGAGC-3’ and 5’-ATATCTCGAGTCAGAGCTTGTGGGGGTTCACCCACTTG-3’);

human *GSTP1* (5’-ATATAAGGATCCATGCCGCCCTACACCGTGGTC-3’ and 5’-AGTGCCTCGAGTCACTGTTTCCCGTTGCCATTG-3’);

Human *GSTP1-D1* (5’-ATATAAGGATCCATGCCGCCCTACACCGTGGTC-3’ and 5’-AAGCGGCCGCTCACAGGATGGTATTGGACTGGT-3’);

Human *GSTP1-D2* (5’-TTAAGCTTCGTCACCTGGGCCGCACCCT-3’ and 5’-TTTGCGGCCGCTCAGCCTCCCTGGTTCTGGGACA-3’)

Human *GSTP1-D3* (5’-GCCCAAGCTTAAGACCTTCATTGTGGGAGA-3’ and 5’-TTTGCGGCCGCTCACTGTTTCCCGTTGCCAT-3’)

### Crosslinking assay

Half gram Epoxy-activated sepharose-6B (GE Healthcare Life Sciences) was swollen in 50 mL distilled water for 1 hour, and 50 ml coupling buffer (0.1 M Na_2_CO_3_, pH 13.0 or pH 9.5) was applied for washing beads through a sintered glass filter. Lactic acid (Sigma-Aldrich) (0.5 g in 15 ml coupling buffer) was mixed with Epoxy-activated sepharose-6B, the pH adjusted to either 9.5 or 13.0, and the coupling allowed for 16 h at 37 °C with gentle shaking. The beads were washed to get rid of free ligand with coupling buffer, and remaining active chemical groups blocked by 1 M ethanolamine at pH 8.0. The amounts of cross-linked ligand were much higher in the 6B-LAC-2 (pH 13.0) than the 6B-LAC-1 (pH 9.5) resins (text). The beads were preserved in a solution containing 20% ethanol until use. Before use, ethanol was washed off, and the beads were incubated with cell lysates for 4 h, washed 4 times with HEPES-lysis buffer (50 mM HEPES-KOH, pH7.4, 180 mM NaCl, 1.5 mM MgCl_2_, 5 mM EDTA, 10% glycerol, 1% NP-40, 100X NaVO_3_), and re-suspended with HEPES-lysis buffer for Western-Blot and mass spectrometry analyses.

### Immunoprecipitation (IP)

For immunoprecipitation, cells were lysed in HEPES-lysis buffer (50 mM HEPES, 150 mM NaCl, 1.5 mM MgCl_2_, 5 mM EDTA, 10% glycerol and 1% Triton X-100, pH7.4) supplemented with a protease inhibitor cocktail (Selleck Chemicals), and then centrifuged at 14,000 g for 15 min. For IP reactions with ectopically expressed and tagged proteins, Flag- (Selleck Chemicals) or HA-beads (MCE) were added to the lysates, stirred at 4 °C for 2 h and washed with HEPES-lysis buffer 4 times. Immuno-precipitated proteins were eluted with Flag or HA peptide (MCE) dissolved in 100 ul HEPES-lysis buffer for 3 h, and the supernatant collected for analyses. For IP reactions with endogenous G6PD, the supernatants were pre-cleared with protein A/G-coupled agarose (Thermo Fisher Scientific), anti-G6PD antibody was added to the cell lysates and stirred at 4 °C overnight, and then protein A/G-coupled agarose were added and incubated for 2 h, followed by washing 4 times and boiling in 5X loading buffer. Immuno-enriched samples were resolved by SDS-PAGE and analyzed by Western-Blot.

### siRNA-mediated RNA Interference

For RNA interference, cells in 6-well plates were transfected with designed siRNAs (an irrelevant sequence [control] siRNA), GSTP1 siRNA-1 and GSTP1 siRNA-2, each at 5 nmol/L, using Lipofectamine RNAiMAX Transfection Reagent (Invitrogen) for 48 h according to the manufacturer’s instructions.

The sequences are:

control *siRNA* (5’- TTCTCCGAACGTGTCACGT-3’);

*siGSTP1*-1 (5’-CATCAATGGCAACGGGAAA-3’);

*siGSTP1*-2 (5’-ACACCGTGGTCTATTTCCCAGTTCG-3’);

*siSRC*-1 (5’-GCCTCAACGTGAAGCACTA-3’);

*siSRC*-2 (5’-GGTGGCCTACTACTCCAAA-3’).

### Cell proliferation assay

Three thousand of MCF-7 cells were seeded with normal media in 12-well plates and switched to glucose-deprived media with varying doses of lactic acid next day. Post specified incubation days, cells were trypsinized, collected, stained with Trypan Blue, and numbers of viable cells counted.

### Colony formation assay

Five hundred cells were seeded in 6 cm plates in triplicates and incubated in specified medium for a total of 14 days with the medium changed once on day 7. Viable colonies were stained with crystal violet.

### NADPH/NADP^+^ ratio measurement

NADPH/NADP^+^ ratio was determined by enzymatic cycling methods as previously described [71], the values were normalized to the concentration of protein.

### Cellular ROS detection assay

After treatment with 0.5 mM glucose with or without 20 mM lactic acid, MCF-7 cells were washed with PBS twice, cultured with the medium with 25 μΜ Η_2_DCFDA probe for 30 min, and the fluorescence intensity detected by excitation at 485 nm and emission at 535 nm.

### Recombinant protein purification and in vitro kinase assay

Full-length human G6PD (wild-type, 2YA or 2YF mutant) sequences were cloned into the pGEX-4T1 vector and expressed as the GST-tagged fusion proteins in *E. coli* BL21 by induction with 0.15 mM isopropyl β-D-thiogalactopyranoside (Sigma Aldrich) for 20 h at 20 °C. The proteins were purified using glutathione-sepharose 4B beads (GE Healthcare Life Sciences). To assess phosphorylation in vitro, substrates GST-G6PD WT, GST-2YF or GST-2YA (1.5 µg) was incubated with recombinant SRC (0.5 µg) for 30 min at 37 °C in a 50 μl kinase reaction system (50 mM Tris-HCl pH 8.0, 150 mM NaCl, 100 mM DTT, 10 mM MgCl_2_) with 0.3 mM ATP. The reactions were terminated by loading buffer, samples resolved by SDS-PAGE and specific signals probed through Immuno-Blot using G6PD p-Tyr and site-specific antibodies.

### G6PD enzyme activity measurement

G6PD enzyme activity was determined following the protocol [72], enzyme activities were normalized to the concentration of protein.

### Lactic acid-responsive tripartite complex assay in vitro

GST-SRC (1.1 μg) protein expressed in bacteria was incubated with GST Sepharose beads for 2 h, washed and resuspended with 100 μl reaction buffer. His-G6PD (1.1 μg) and His-GSTP1 protein (1.1 μg) were incubated with GST-SRC beads in the reaction buffer (20 mM HEPES, pH 7.5, 100 mM NaCl) overnight with or without 20 mM lactic acid, washed for four times and analyzed by Western Blotting, which were stained by Coomassie Brilliant Blue.

### GSTP1 activity measurement

Cells treated with or without 20 mM lactic acid were sonicated on ice and centrifuged. The supernatant was mixed with 160 μl solution II and 20ul solution III provided in the kit (Solarbio) and the absorbance at 340 nm measured at the interval of 30 s for 10 min. The activity of GSTP1 was calculated.

### Lactate measurement

Briefly, three kinds of beads were collected for lactate measurement. The master reaction mix contained 5 ml reaction buffer (0.1 M Tris-HCl, pH8.6), 1 ml cosolvent (3% Triton X-100), 2 ml chromogenic reagent (120 mg Nitrotetrazolium blue chloride, 4 mg Phenazine methosulfate and 250 mg NAD^+^ dissolved in 50 ml sterilized water) and 130U LDH were prepared. For each well of 96-well plate, 175 µl reaction buffer and 25 μl beads were mixed and incubated at 37 °C for 30 min. The absorbance was measured at 570 nm (A570) on SpectraMax M5. The concentrations of the lactate used to generate the standard curve were 0, 0.0983, 0.1875, 0.375, 0.75 and 1.5 mM.

### Dot blotting

A serial dilution with 0.5 pg, 0.005 ng, 0.05 ng, 0.5 ng and 1 ng of Y249(pi) or 322(pi) peptides along with unmodified peptides (HuaAn Biotechnology) were spotted onto nitrocellulose membranes. After complete absorption of the samples, the membranes were blocked with 5% skim milk for 1 h and incubated with Y249(pi) or Y322(pi) antibodies for 2 h at room temperature, followed by 1-hour incubation with a secondary antibody that was conjugated with horseradish peroxidase (HRP). After washing, HRP substrate was added to develop the images captured and recorded by exposure to X-ray films.

### Simulation of GSTP1-Lactate and GSTP1-G6PD-SRC complex

The structure of the GSTP1 protein (PDB ID: 3GUS)was downloaded from the PDB website and small molecules removed through Pymol. The molecular structure of lactic acid was extracted from the structure of the protein (PDB ID: 3KB6). Autodock tools were used to simulate the interaction between GSTP1 and lactate and calculate the most possible binding energy. In addition, GSTP1, G6PD (PDB ID: 2bhl) and SRC (PDB ID: 2H8H) structures were each extracted as above and were modeled by GRAMM-X Protein-Protein Docking Web.

### Quantification of metabolites by LC-MS/MS

To quantify polar metabolites concentrations, we used TSQ Vantage LC-MS interfaced with Ultimate 3000 Liquid Chromatography system (Thermo Scientific) and Triple quadruple mass spectrometry to detect metabolites from tissues or cells. Polar metabolites were extracted according to a protocol. Briefly, we added 80% HPLC-grade methanol (cooled to −80 °C) to fresh or frozen tissues or cells that were grinded for 1–2 min with tissue grinder in the tube on dry ice. After vortexing for 1 min at 4–8 °C and incubated for 4 h at −80 °C, the samples were then centrifuged at 14,000 g for 10 min at 4 °C. We transferred the supernatant to a new 2 ml tube and lyophilize the samples in a speedVac prior to LC-MS analysis. For quantification of metabolites, U-13C-glutamine was added to the extraction buffer in 80% methanol and used as internal standard. Samples and standards were measured using a TSQ Vantage equipped with a HILIC column (Amide 4.6 x 100 mm ID 3.5 μm; Part No: 186004868, Waters). The mobile phases and gradients were the same as those used for the sugar phosphate on the UHPLC-QTOF system (AB 6600 TripleTOF, SCIEX, Canada). The ion transfer tube temperature was set to 350 °C and vaporizer temperature was 270 °C. The instrument was run in the negative mode with a spray voltage of 3000, sheath gas 40 and Aux gas 5.0. The 7–8 concentrations (from low to high) of the different standard mix were measured using multiple reactions monitoring mode (MRM) with optimal collision energies to produce a standard curve.

### Mice

All animal procedures were conducted with the approval of the Animal Research Ethics Committee at Zhejiang University. All animals were taken care of in a humane way following standard guidance information of the Care and Use of Laboratory Animals written by NIH. Female BALB/c nude mice (3 weeks old) in good conditions were purchased from GemPharmatech Company in Jiangsu, China. All the mice were housed in Laboratory Animal Center of Zhejiang University with stable temperature (23 ± 2 °C) and light/dark at the interval of 12 h.

### Xenograft mouse models

Mice were injected subcutaneously with 1 × 10^7^ stable cells (*n* = 6 for each group). At day 15, when tumors became visible, tumor growth was measured by caliper every 2 or 3 days, tumor volume was calculated as the formula of 1/2× major diameter × minor diameter^2^. When the tumor reached 100–150 mm^2^, mice were divided into three groups following the principle of randomness and respectively received subcutaneous Intraperitoneal injection every two days with Tween80 (vehicle control, 100 μl), GSPT1 inhibitor (HY135318; MCE) (5 µM and 10 µM in 100 μl of Tween80, PEG300 and saline solution). Mice were sacrificed when growth retardation of tumors was evident or when tumors reached the maximally permitted condition. After dissection, the weights of tumors were measured and recorded.

### Statistical analysis

Statistical analyses were conducted on GraphPad Prism 7 or with appropriate computational tools. The data were analyzed with unpaired students’ t-test, multiple t test or two-way analysis of variance (ANOVA) as indicated in the figure legends. And the variation was estimated in every test. Error bars show sampling bias from independent samples or experiments. The correlation analyses were conducted by Pearson’s test. All data including the Western-blots were from at least two independent experiments with SEM (mean ± SD). ns, not significant, *p < 0.05, **p < 0.01, ***p < 0.001, ****p < 0.0001. For animal models, we underwent a randomization process in mice within the same groups. Original western blots can be obtained in the “Supplementary Material” file.

## Supplementary information

checklist

Agreement letter

ORCID

supplementary figure legends

Figure S1

Figure S2

Figure S3

Figure S4

Figure S5

Figure S6

Figure S7

raw WB data

raw supplementary data

## Data Availability

All data in the main text or the supplementary information are available from the corresponding author upon reasonable request.
