# Supplementary figures and images for: A GSTP1-mediated lactic acid signaling promotes tumorigenesis through the PPP oxidative branch
Source: Cell Death Dis. 2023 Jul 25;14(7):463. doi: 10.1038/s41419-023-05998-4 (PMC10368634; doi:10.1038/s41419-023-05998-4)

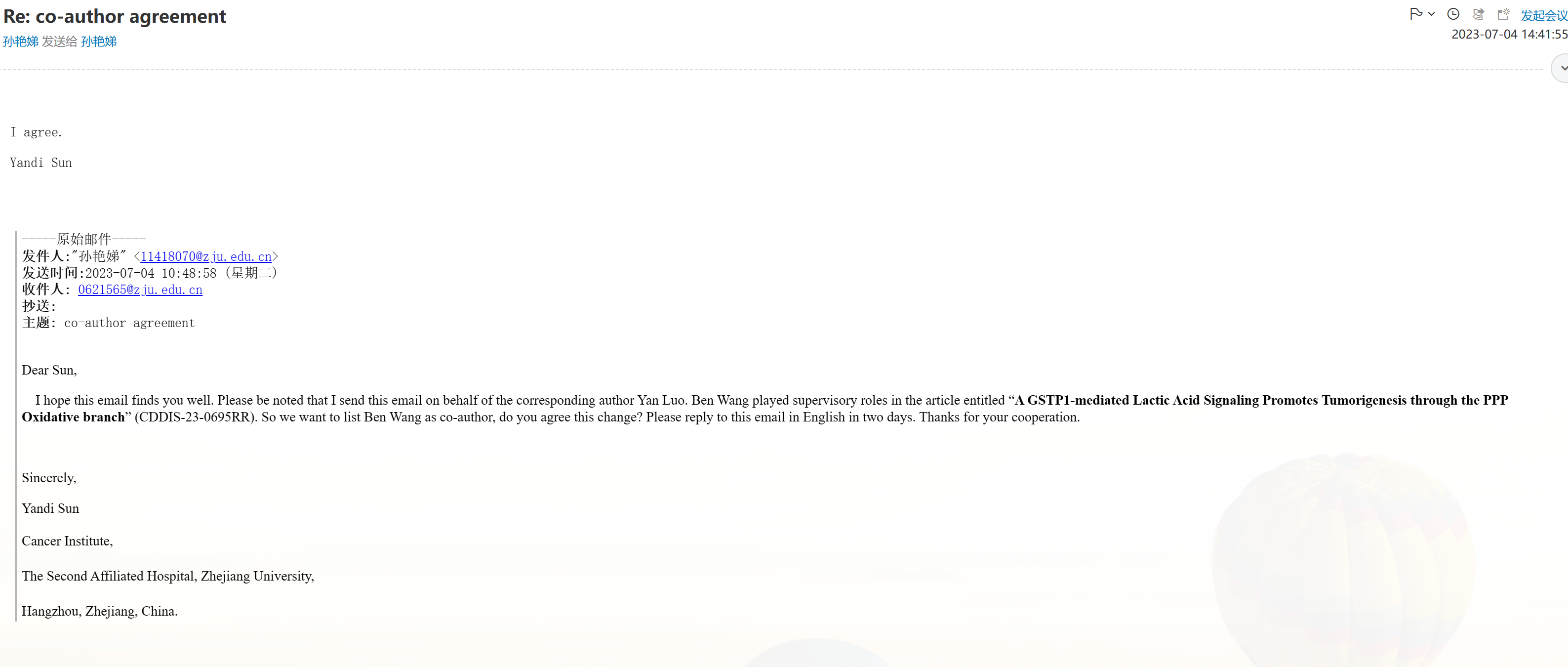

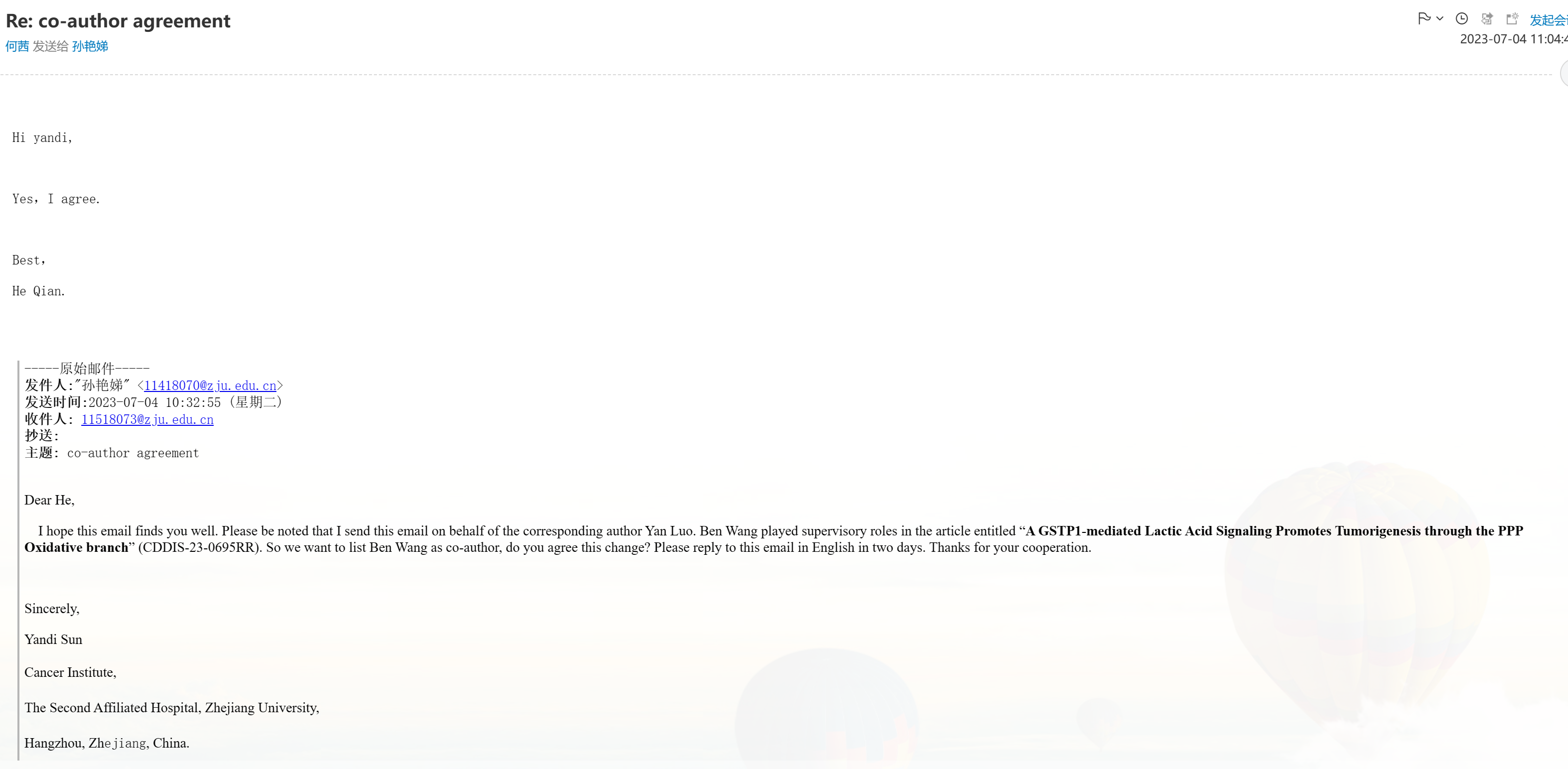


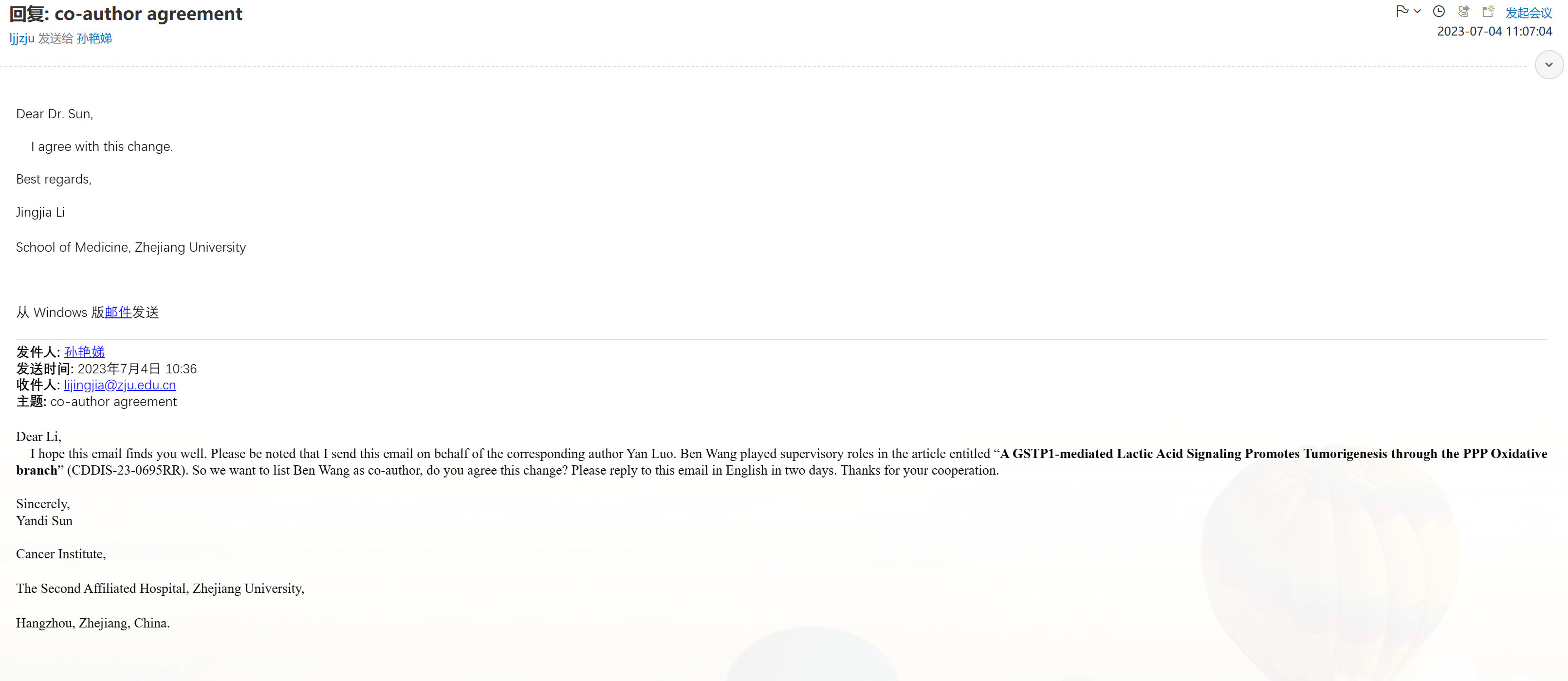

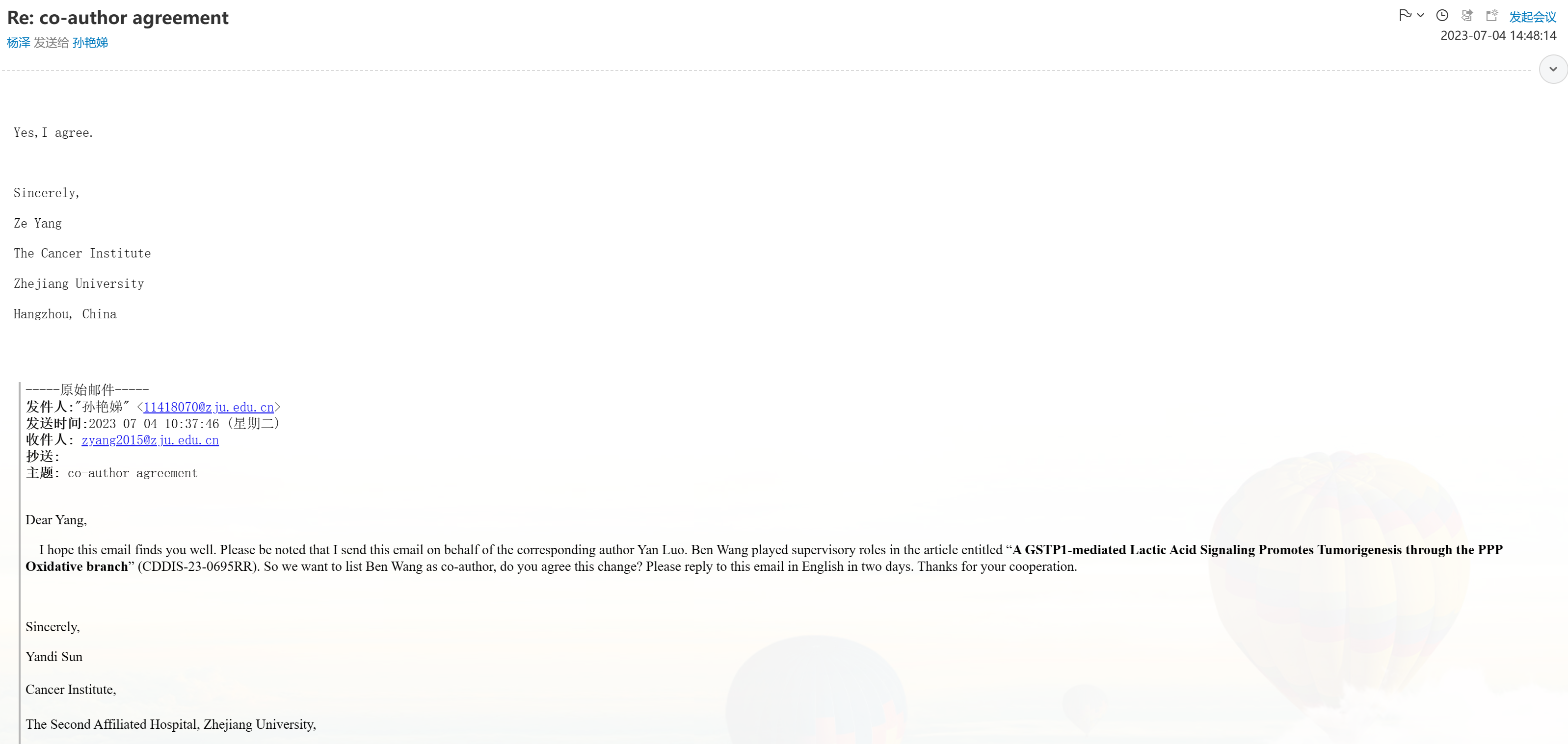

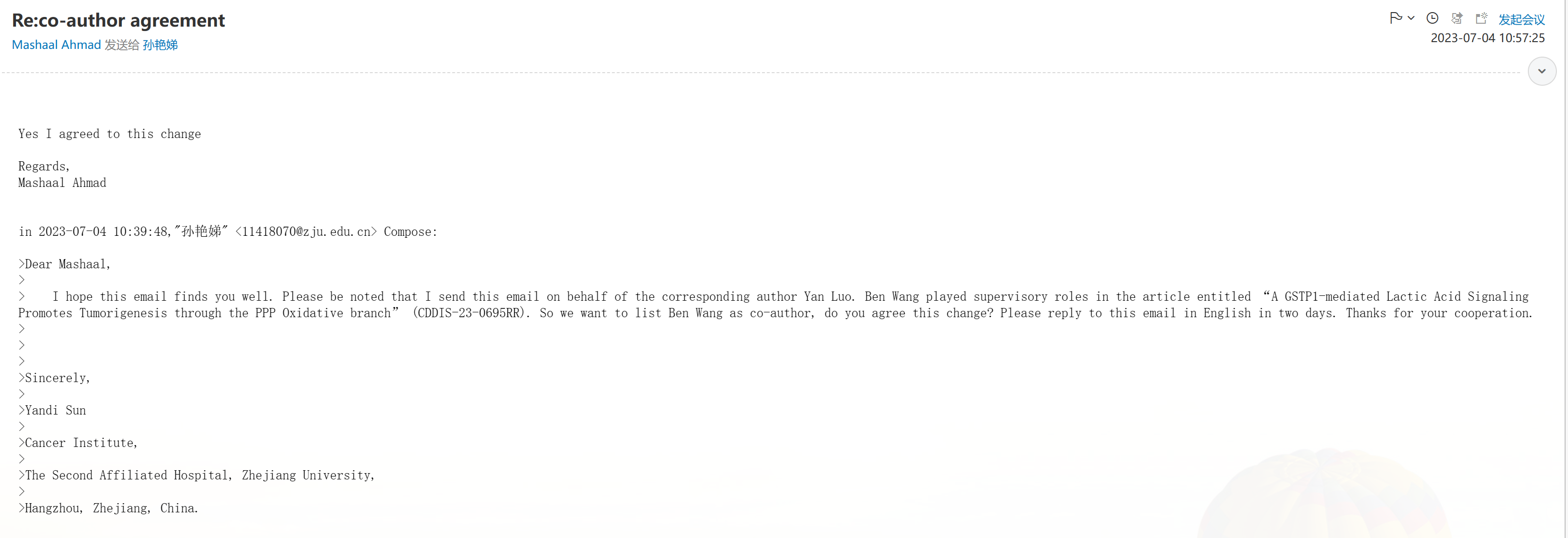

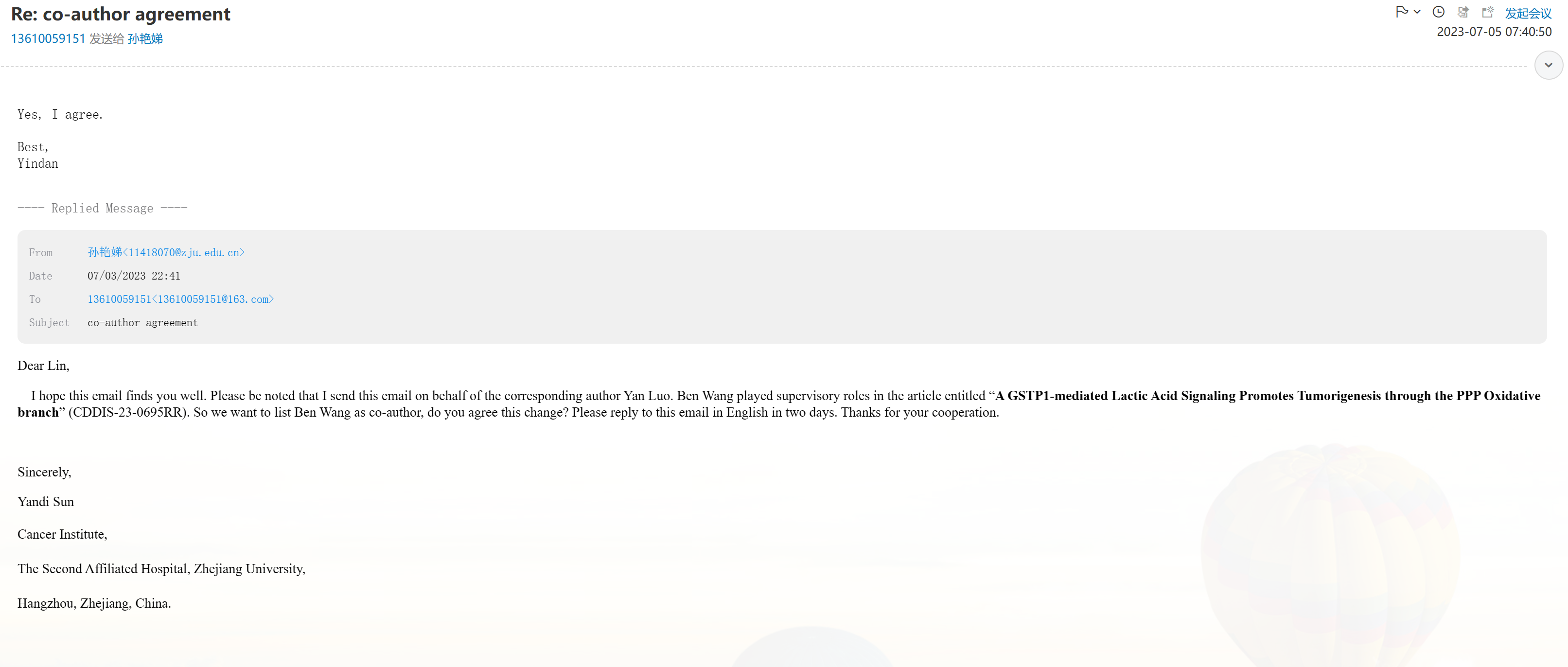

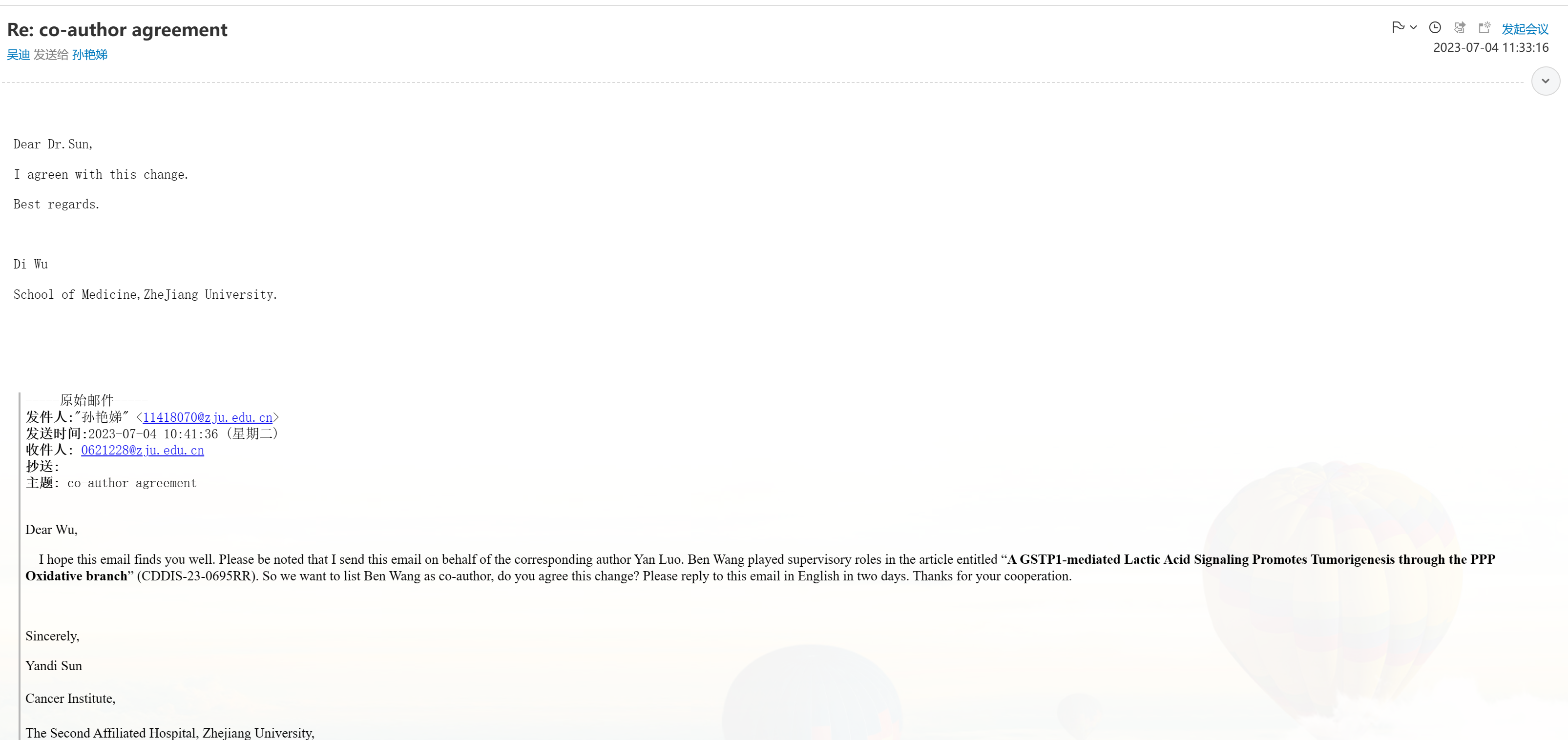

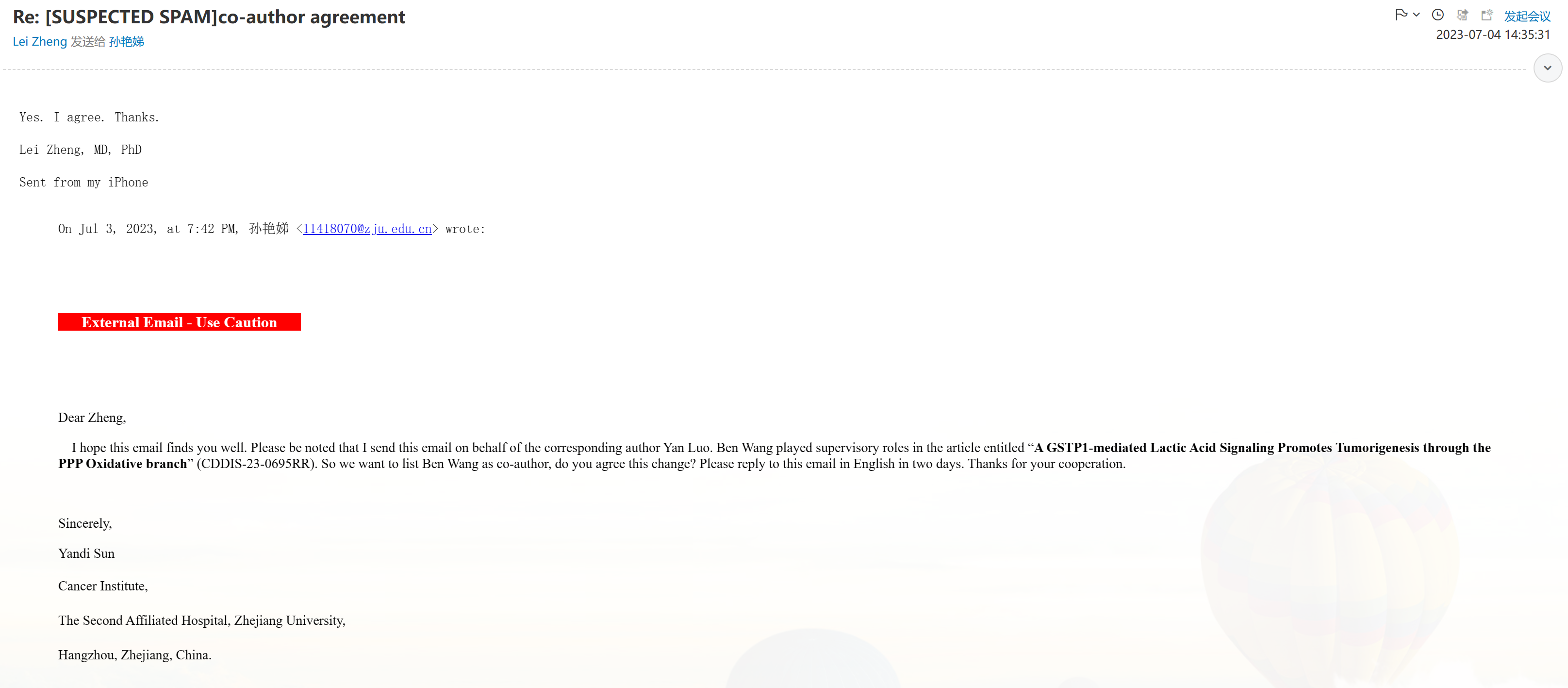

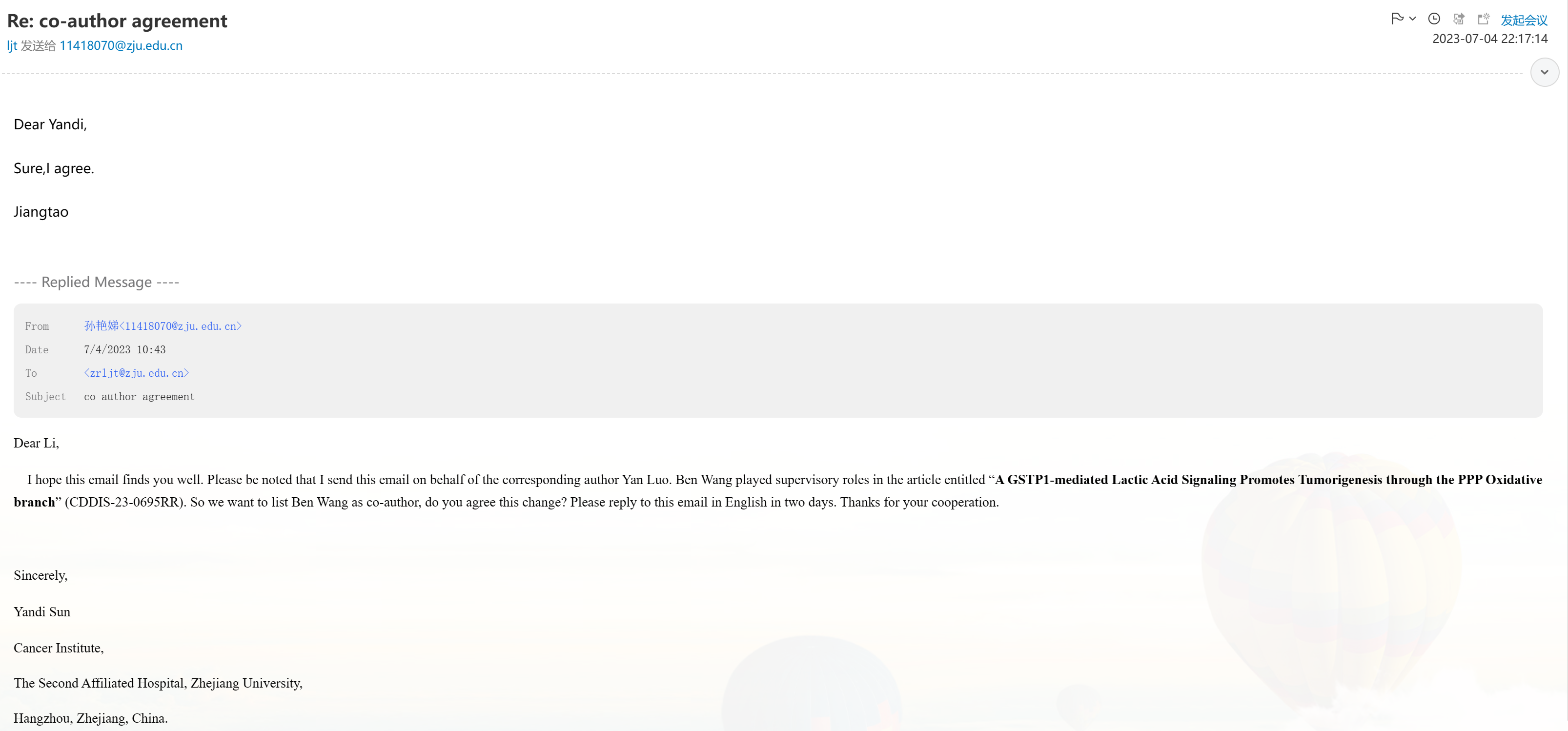

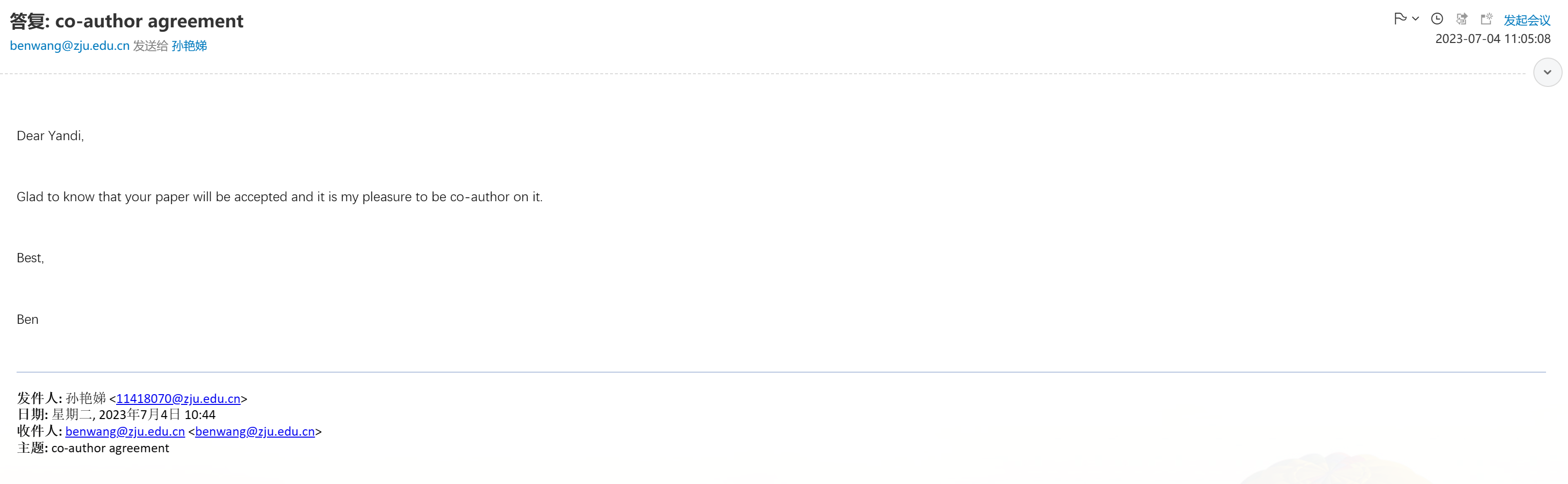

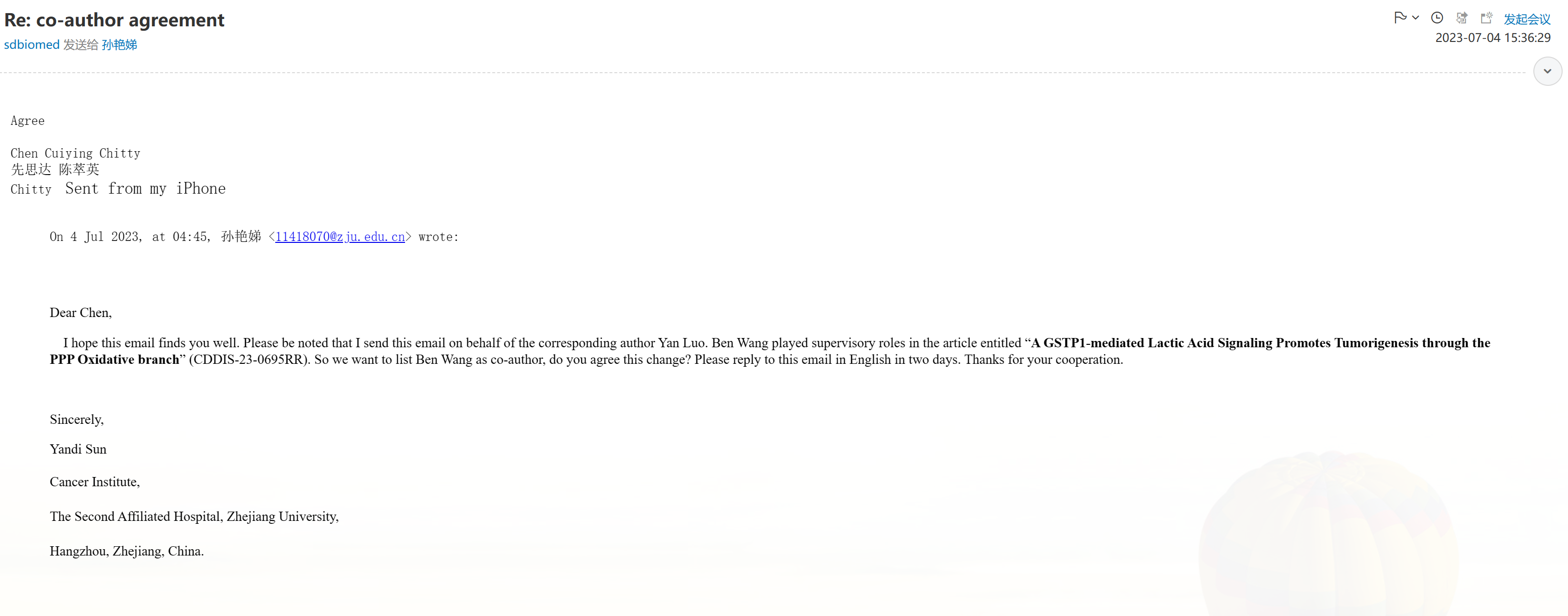

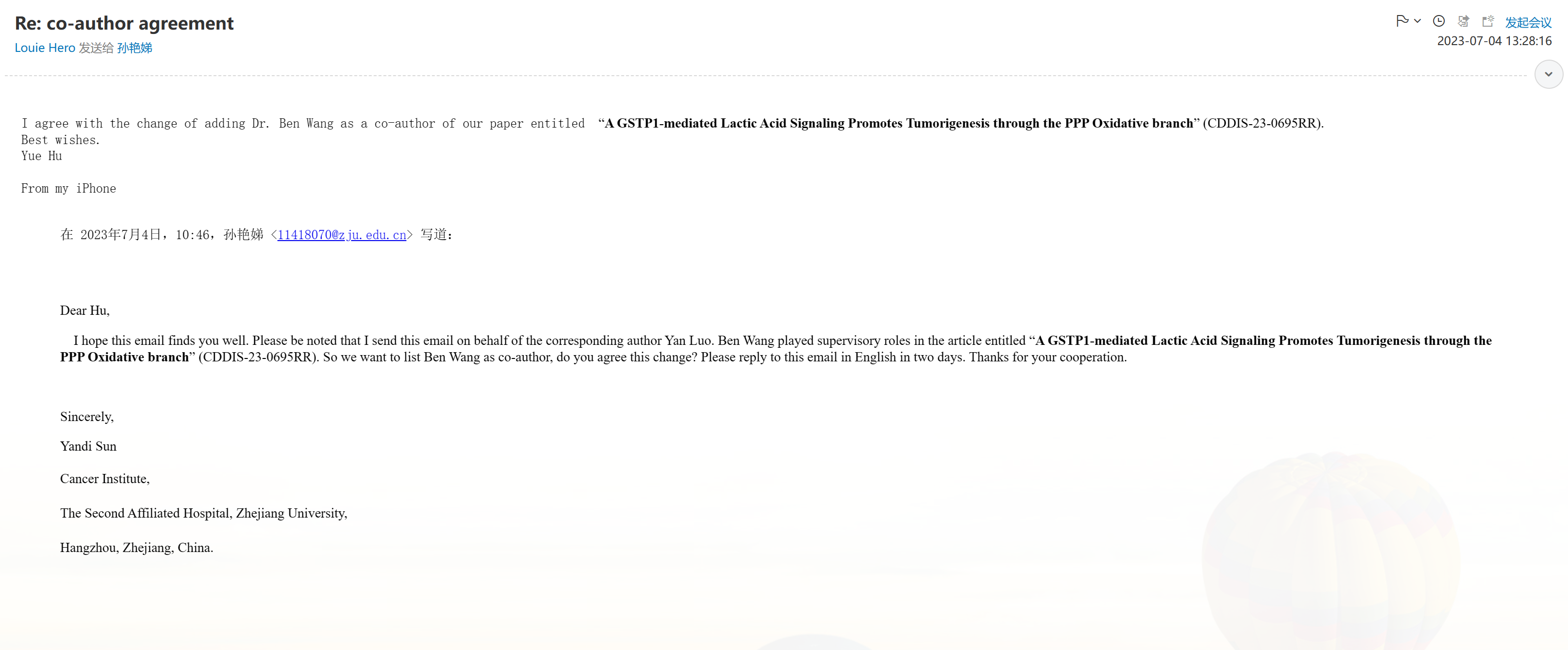

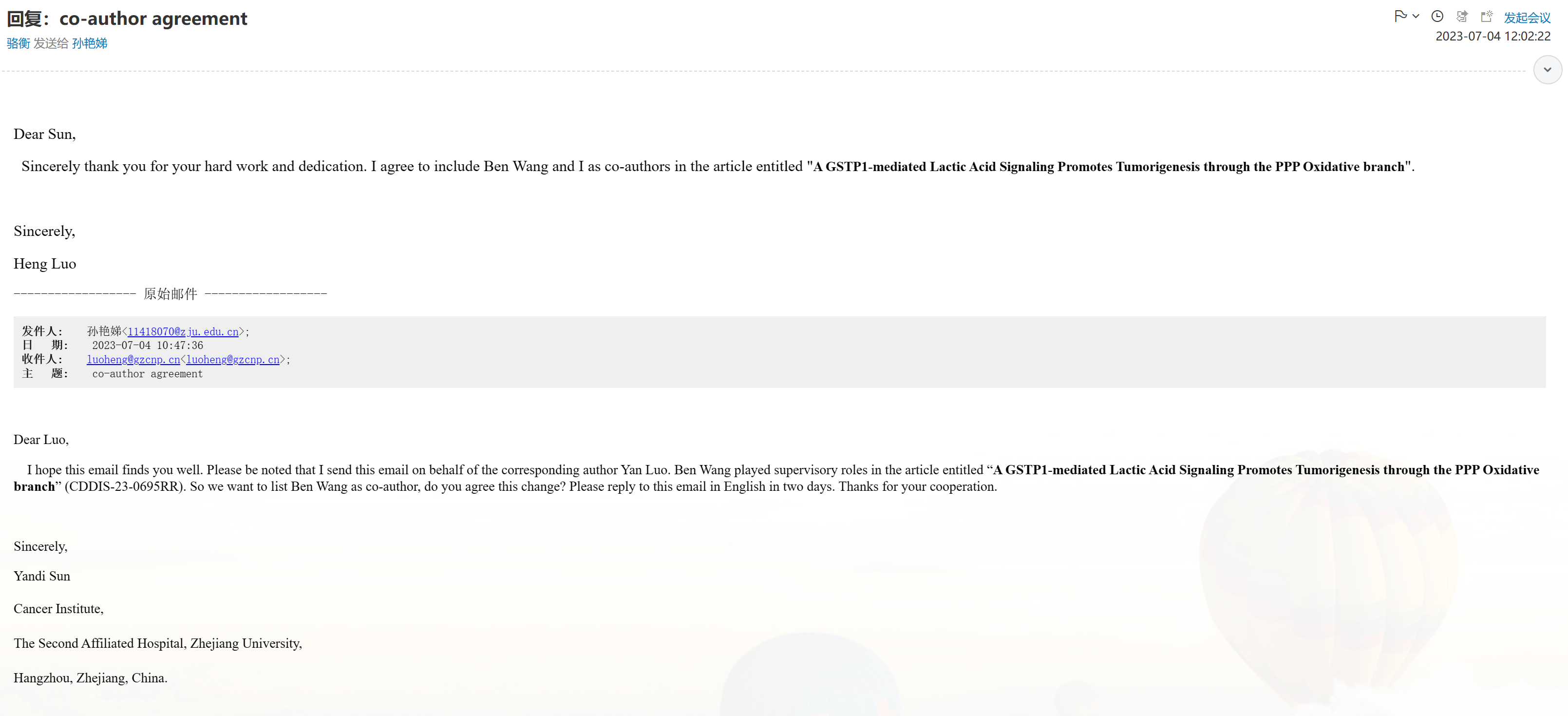

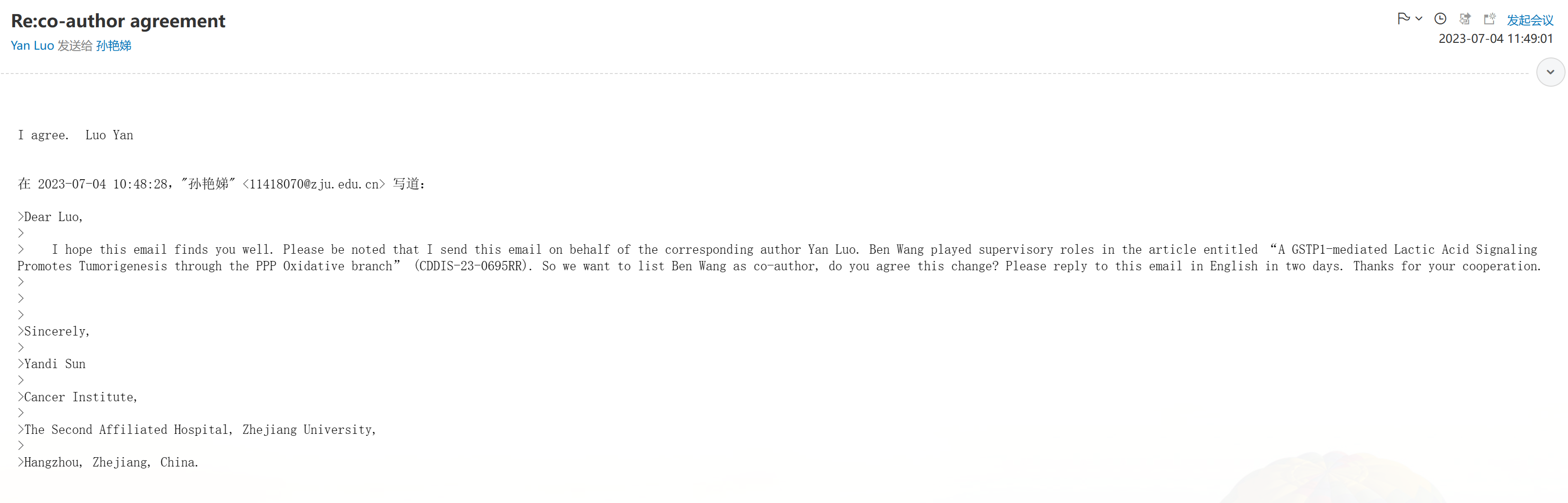

Supplement: Supplementary file 2 — Agreement letter [file 41419_2023_5998_MOESM2_ESM.docx]

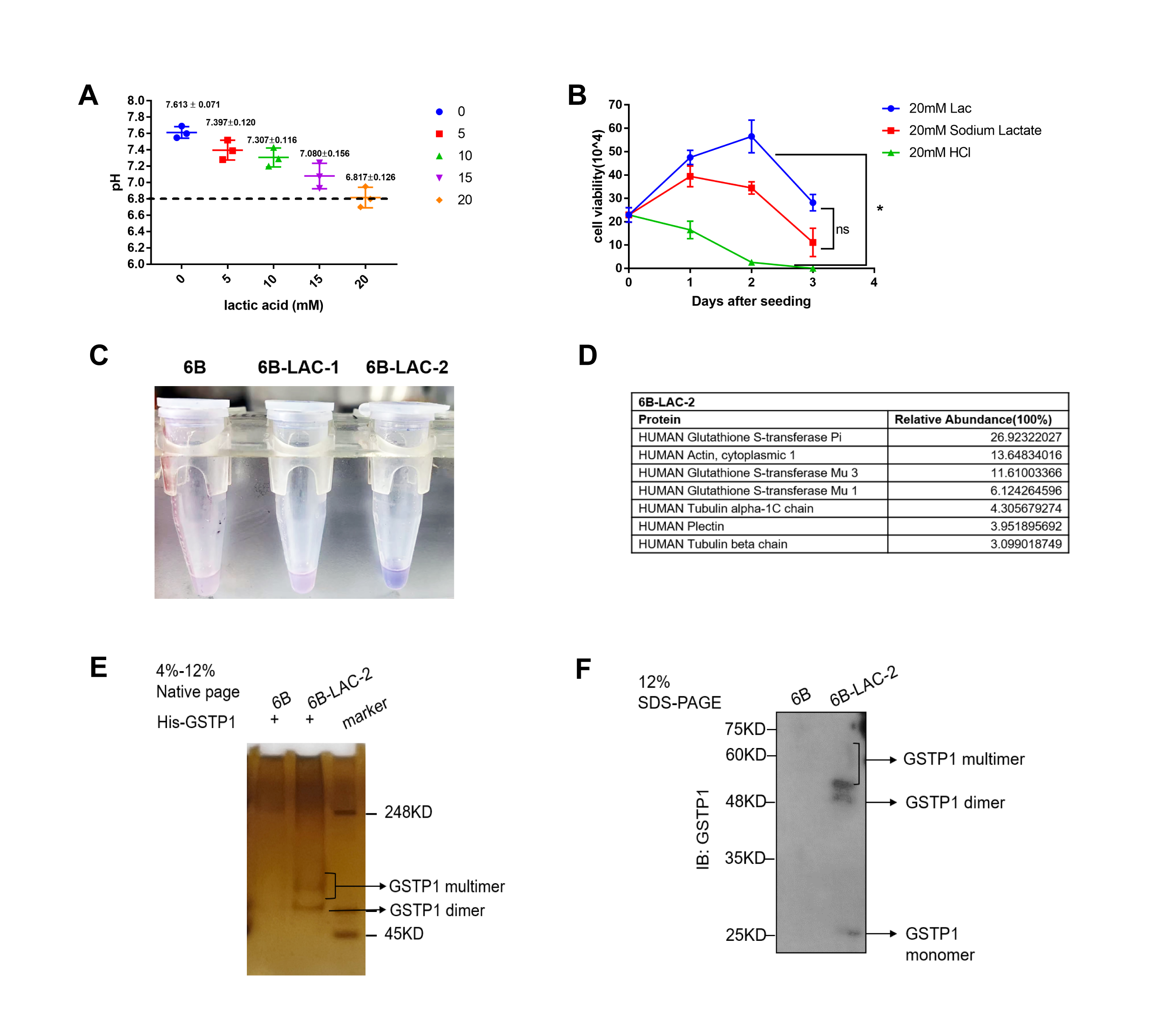

Supplement: Supplementary file 5 — Figure S1 [file 41419_2023_5998_MOESM5_ESM.png]

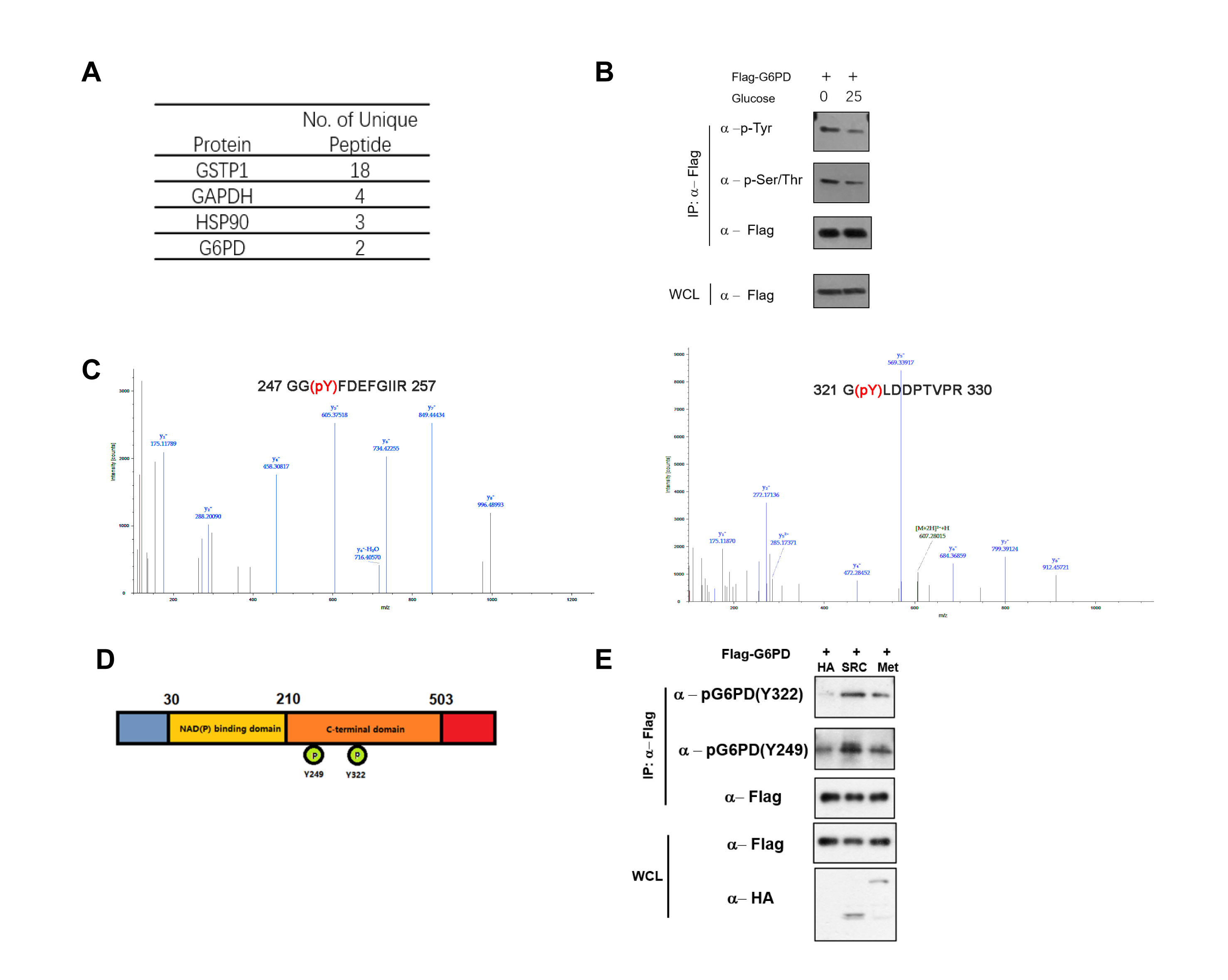

Supplement: Supplementary file 6 — Figure S2 [file 41419_2023_5998_MOESM6_ESM.png]

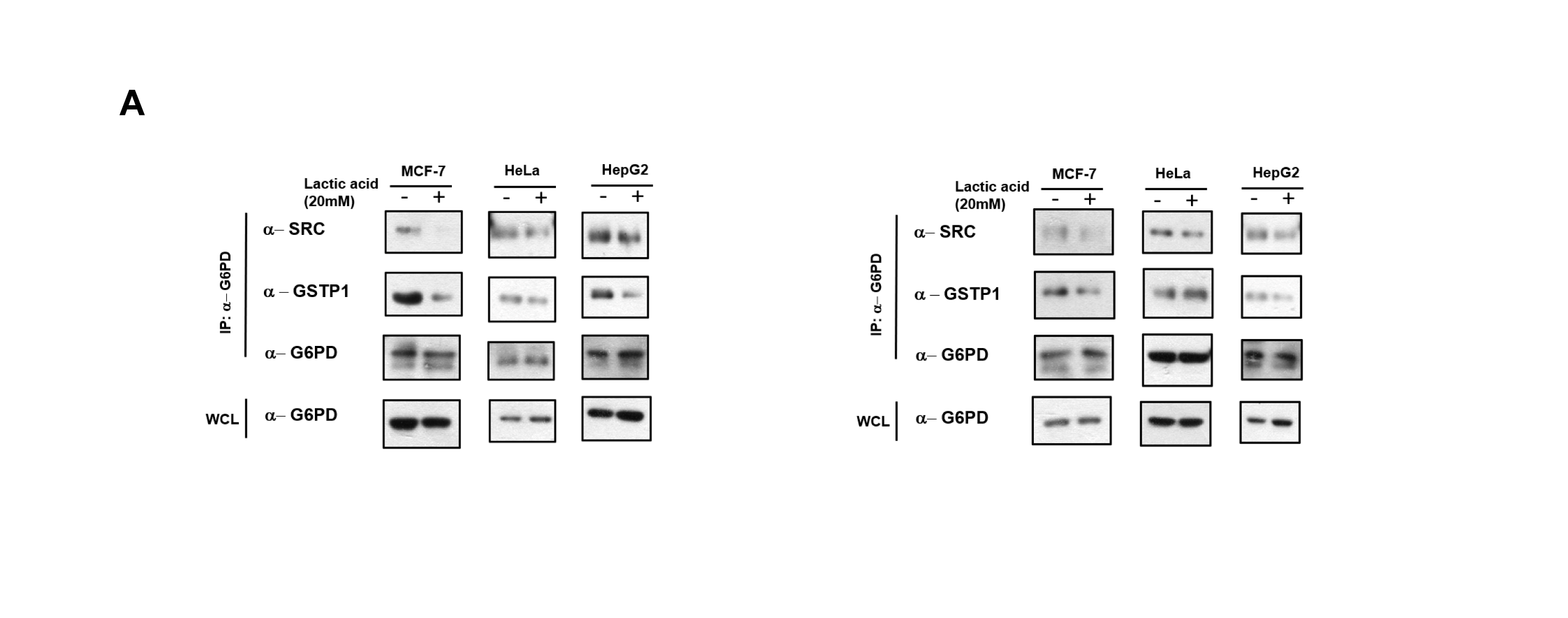

Supplement: Supplementary file 7 — Figure S3 [file 41419_2023_5998_MOESM7_ESM.png]

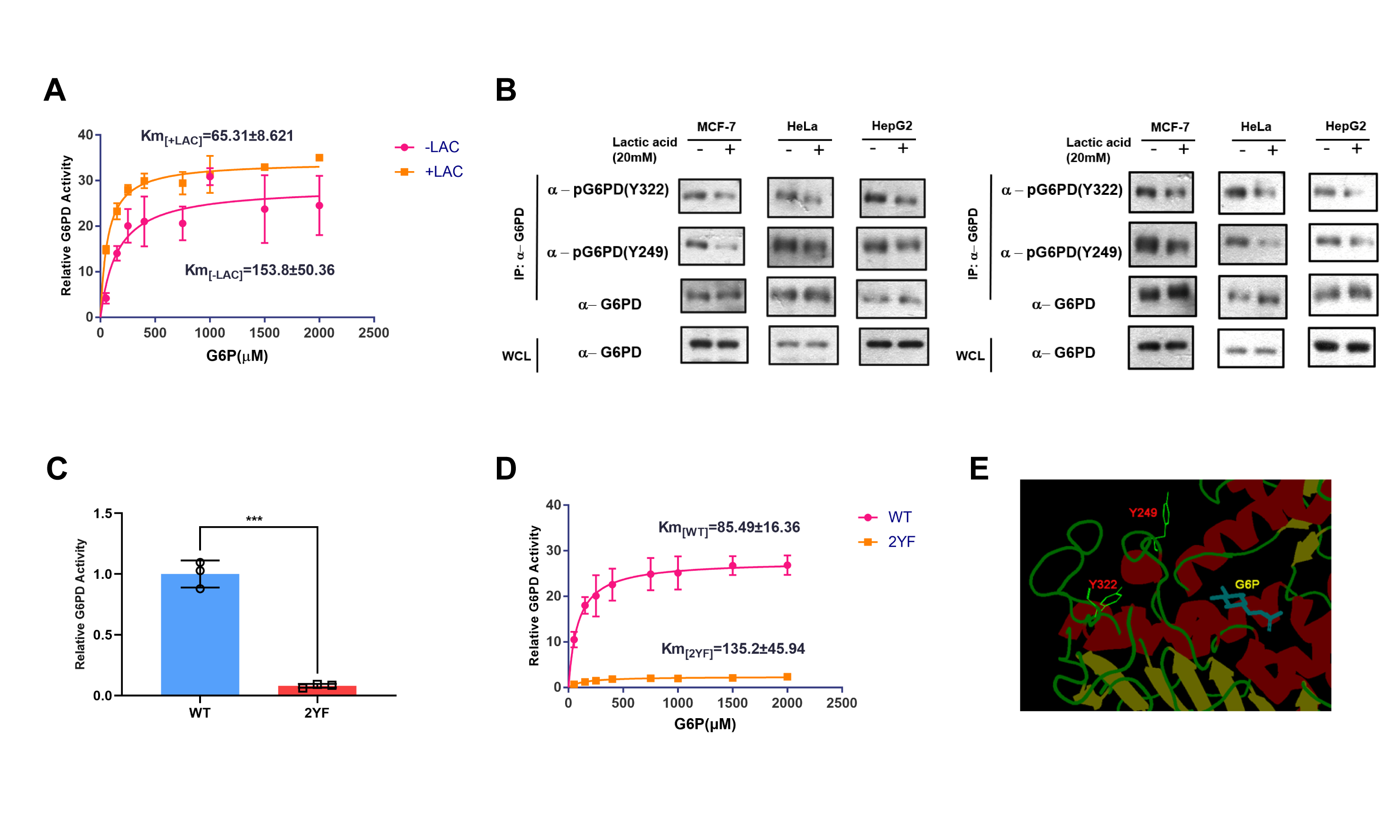

Supplement: Supplementary file 8 — Figure S4 [file 41419_2023_5998_MOESM8_ESM.png]

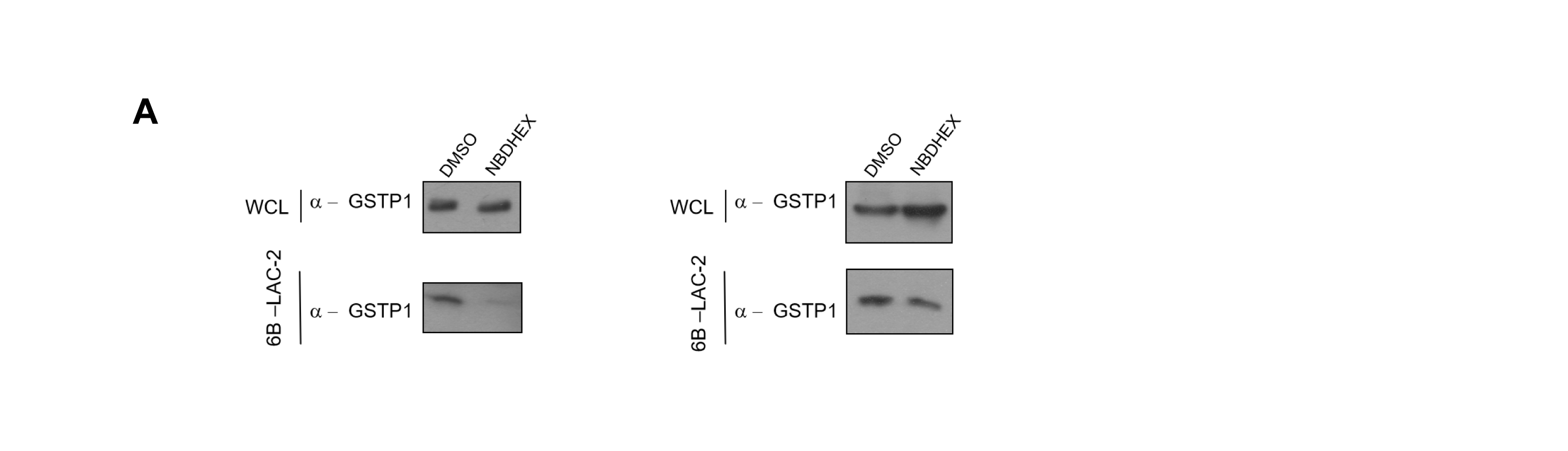

Supplement: Supplementary file 9 — Figure S5 [file 41419_2023_5998_MOESM9_ESM.png]

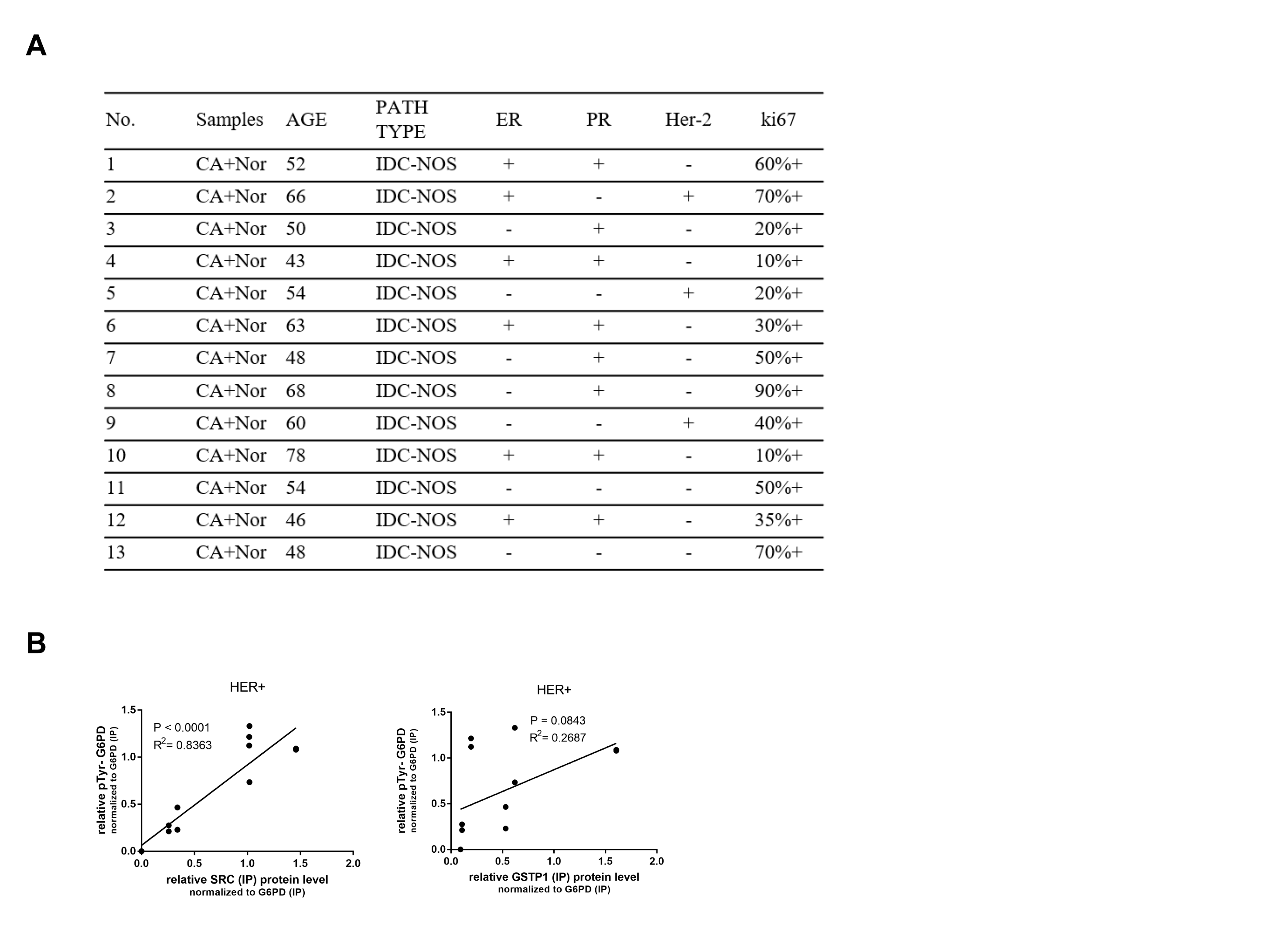

Supplement: Supplementary file 10 — Figure S6 [file 41419_2023_5998_MOESM10_ESM.png]

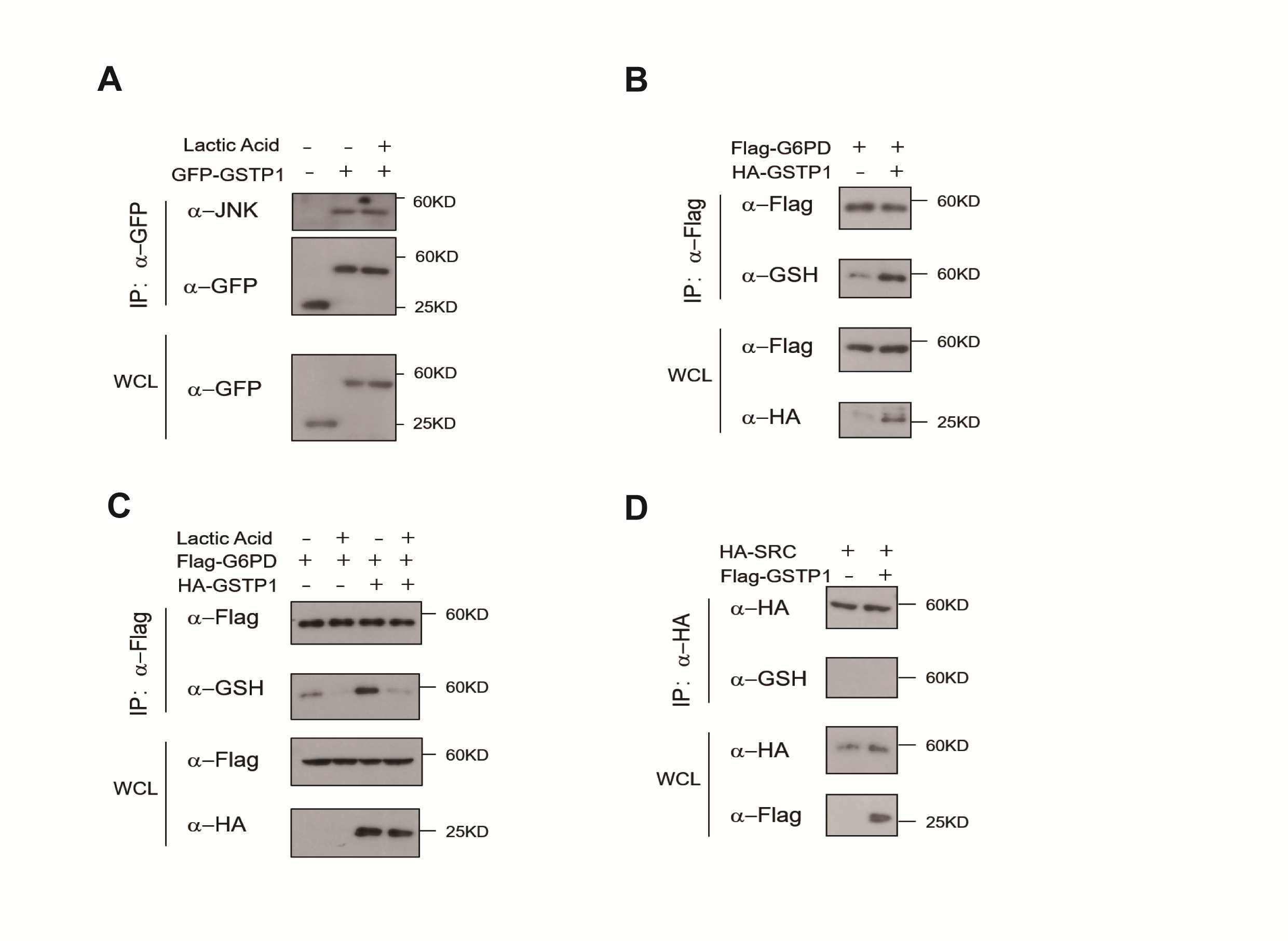

Supplement: Supplementary file 11 — Figure S7 [file 41419_2023_5998_MOESM11_ESM.png]
