# Supplementary material for: A GSTP1-mediated lactic acid signaling promotes tumorigenesis through the PPP oxidative branch
Source: Cell Death Dis. 2023 Jul 25;14(7):463. doi: 10.1038/s41419-023-05998-4 (PMC10368634; doi:10.1038/s41419-023-05998-4)
Supplement: Supplementary file 3 — ORCID [file 41419_2023_5998_MOESM3_ESM.docx]

ORCID of each corresponding author：

Yan Luo：0000-0002-3812-8814

Heng Luo：0000-0003-3418-5739

Yue Hu: 0000-0002-6209-9144
