# Supplementary material for: A GSTP1-mediated lactic acid signaling promotes tumorigenesis through the PPP oxidative branch
Source: Cell Death Dis. 2023 Jul 25;14(7):463. doi: 10.1038/s41419-023-05998-4 (PMC10368634; doi:10.1038/s41419-023-05998-4)
Supplement: Supplementary file 4 — supplementary figure legends [file 41419_2023_5998_MOESM4_ESM.docx]

**Supplementary Figure Legends**

**Figure S1.** (A) The pH values of the media containing 0.5mM glucose with a titration of lactic acid (0-20 mM) were monitored. (B) The growth curve of MCF-7 cells cultured in glucose-deprived (0.5 mM) media with 20 mM lactic acid, 20 mM sodium lactate and 20 mM HCl for 3 days. The pH with the 20 mM HCl medium was adjusted to that with 20 mM lactic acid. p(20mM lac vs 20mM sodium lactate) = ns, ***p(20mM lac vs 20mM HCl) =0.0006. (C) Three types of beads were stained with as mentioned in Methods and Materials, and colors are shown. (D) Relative abundances of proteins eluted from the 6B-Lac-2 resin were determined by quantitative mass spectrometry. (E) The modified 6B beads were incubated with His-GSTP1 from E.Coil for 2 hours and analyzed by 4%-12% Native page. (F) The modified 6B beads were incubated with whole cell lysate for 2 hours and washed by HEPES lysis buffer four times. Then 100µl washing buffer containing 0.03125% glutaraldehyde was added in the resin for 15 min, which was analyzed by Western blotting.

**Figure S2.** (A) The amounts of protein unique peptides immunoprecipitaed with GSTP1 were determined by mass spectrometry. (B) Flag-tagged G6PD protein is Tyr-phosphorylated. MCF-7 cells (human breast cancer) were transfected with Flag-G6PD for 24 hours, switched to glucose-deprived and glucose-rich (25mM) media, and cell lysates were immunoprecipitated for Western-blot analyses. (C) Mass spectrometry data of G6PD phosphorylation sites. (D) A schematic diagram of G6PD phosphorylation sites located in the G6PD protein. (E) SRC protein elevates Flag-tagged G6PD phosphorylation more significantly than c-Met protein. HA-SRC or HA-c-Met plasmids were co-expressed with Flag-tagged G6PD in MCF-7 cells for 16 hours, and M2-enriched Flag-tagged G6PD was immunoblotted with antibodies.

**Figure S3.** (A) Two other immunoblots that emphasize the lactic-acid-modulated tripartite complex formation, which supplements the ones discussed in text.

**Figure S4.** (A) The Km values [without (-lac) and with (+lac) lactic acid] of Flag-G6PD for glucose-6-phosphate (G6P) were determined. Relative enzyme activities were shown as mean ± SD of triplicate experiments. (B) Two other immunoblots that emphasize the lactic-acid-modulated G6PD phosphorylation, which supplements the ones discussed in text. (C) The catalytic activities of immuno-purified G6PD WT vs. Tyr phosphorylation Y249 and Y322 mutant (2YF) protein were determined. Results are mean ± SD of triplicate experiments. The p value was analyzed by Student’s t test. **p=0.009. (D) The Km (for G6P) of the G6PD WT vs. Tyr phosphorylation Y249 and Y322 mutant (2YF) was determined. Relative enzyme activities were shown as mean ± SD of triplicate experiments. (E) In silico modeling of the G6PD catalytic center identifies the two Tyr residues （Y249 and Y322）to be spatially close to the substrate binding pocket.

**Figure S5.** (A) Two other immunoblots emphasizing that NBDHEX reduces the interaction between GSTP1 and 6B-LAC-2, which supplements the ones discussed in text.

**Figure S6.** (A) The information of breast cancer patients. (B) A possible correlation between relative SRC/GSTP1 (IP) levels and relative p-Tyr levels of G6PD was determined in various types of breast cancer samples. Data are analyzed and shown by Pearson Correlation Coefficient. HER+: 3 pairs.

**Figure S7.** (A) MCF-7 cells were transfected with GFP-GSTP1 for 24 hours, switched to glucose-deprived and lactic acid (20mM) media, and cell lysates were immunoprecipitated for Western-blot analyses. (B/C) HA-GSTP1 plasmid was co-expressed with Flag-tagged G6PD in MCF-7 cells, and M2-enriched Flag-tagged G6PD was immunoblotted with GSH antibody under glucose deprivation (B) or lactic acidosis (C). (D) Flag-GSTP1 plasmids were co-expressed with HA-SRC in MCF-7 cells, and the immune-HA-tagged G6PD was immunoblotted with GSH antibody under lactic acidosis.
