# Supplementary material for: A GSTP1-mediated lactic acid signaling promotes tumorigenesis through the PPP oxidative branch
Source: Cell Death Dis. 2023 Jul 25;14(7):463. doi: 10.1038/s41419-023-05998-4 (PMC10368634; doi:10.1038/s41419-023-05998-4)

Figure 1

1F

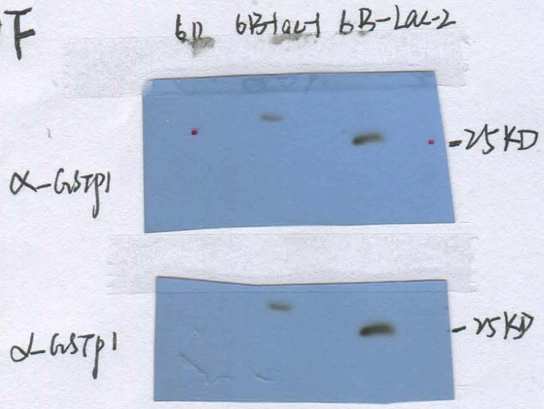

1J

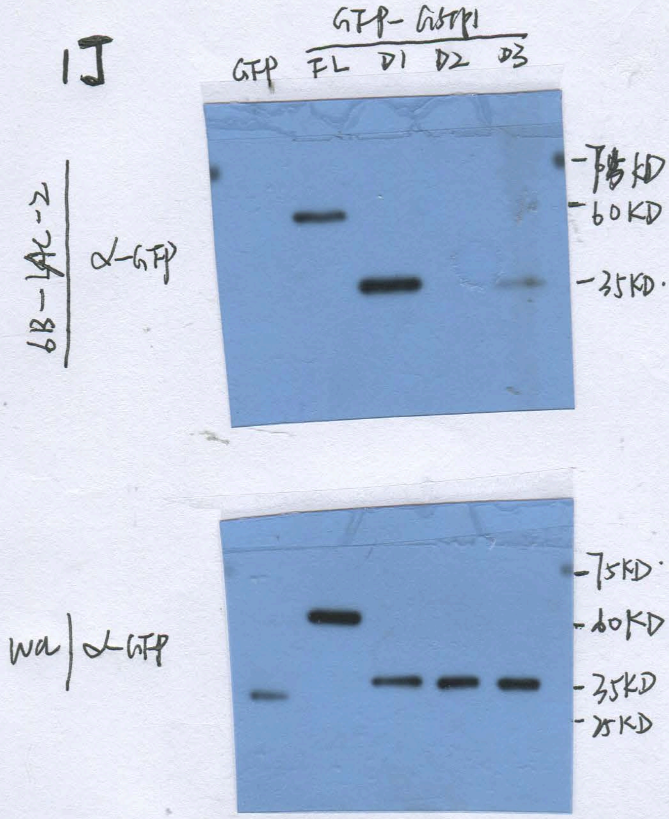

Figure 2

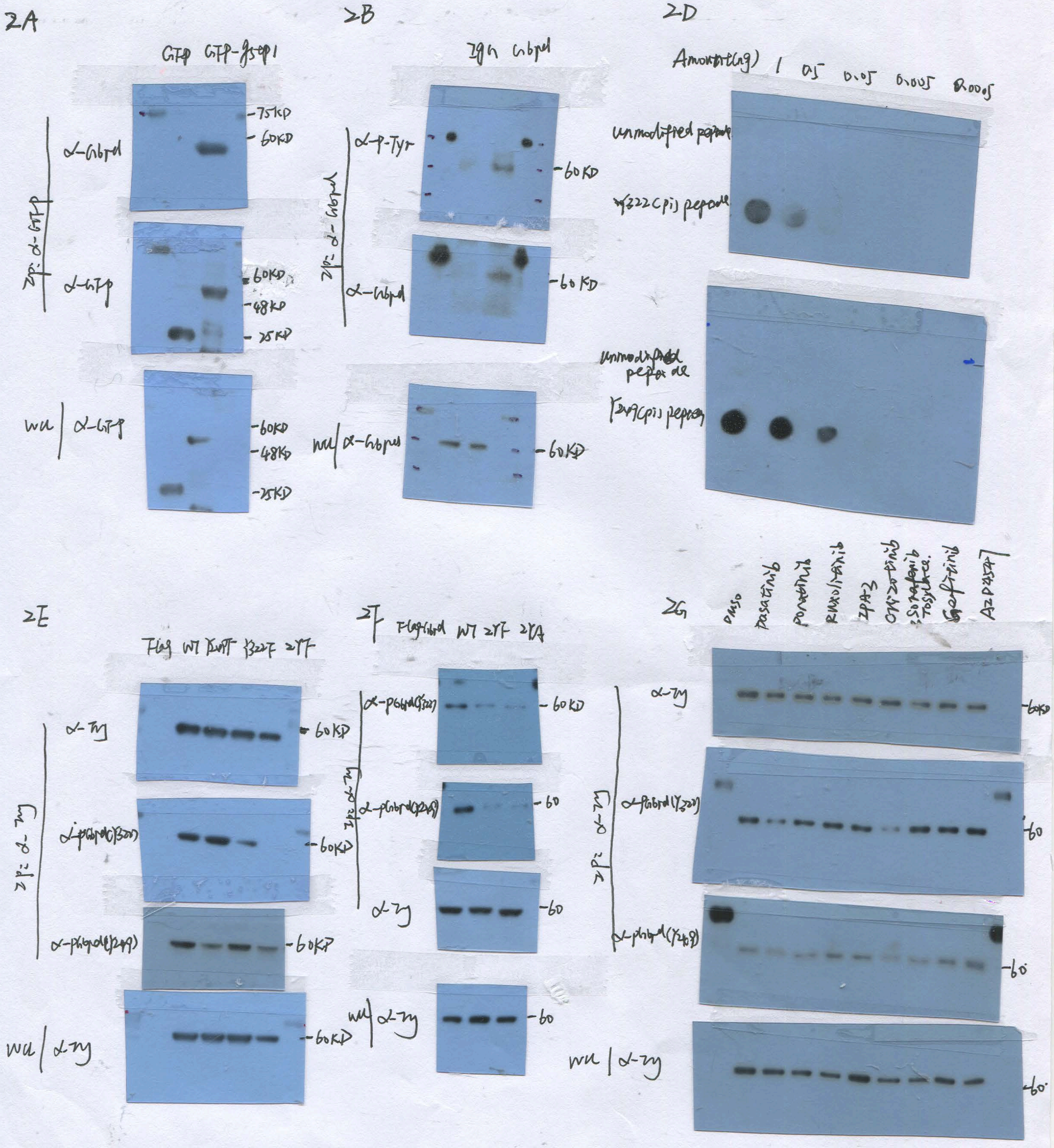
2A
GFP
α-GFP
IP: α-GFP
WCL
75KD
60KD
48KD
25KD
2B
IgG
α-P-Tyr
60KD
2D
Amount(ng)
1
0.5
0.05
0.005
0.0005
unmodified peptide
2E
Flag
WT
α-Myc
60KD
2F
2G
DMSO
Dasatinib
Ponatinib
Ruxolitinib

Figure 2

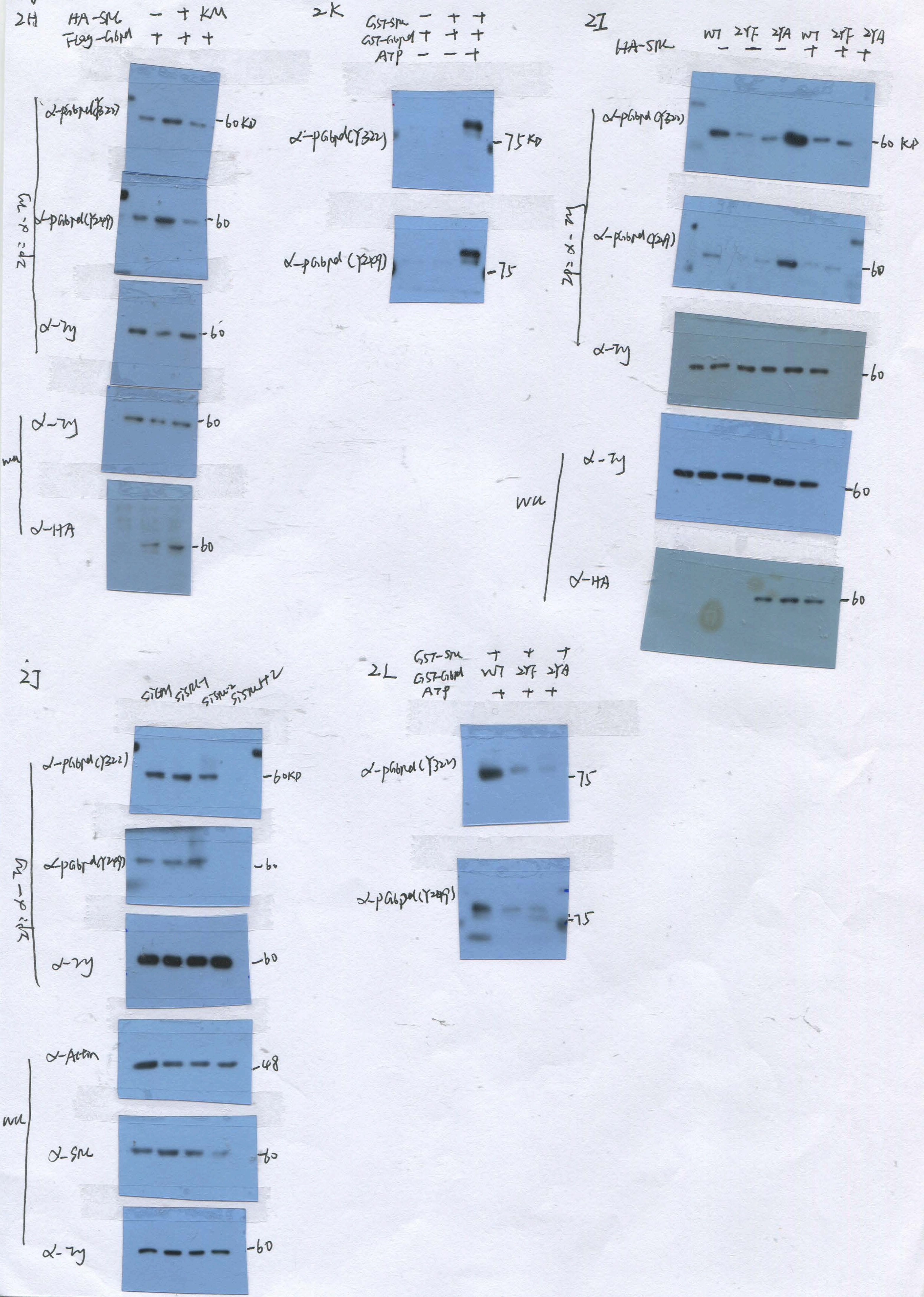

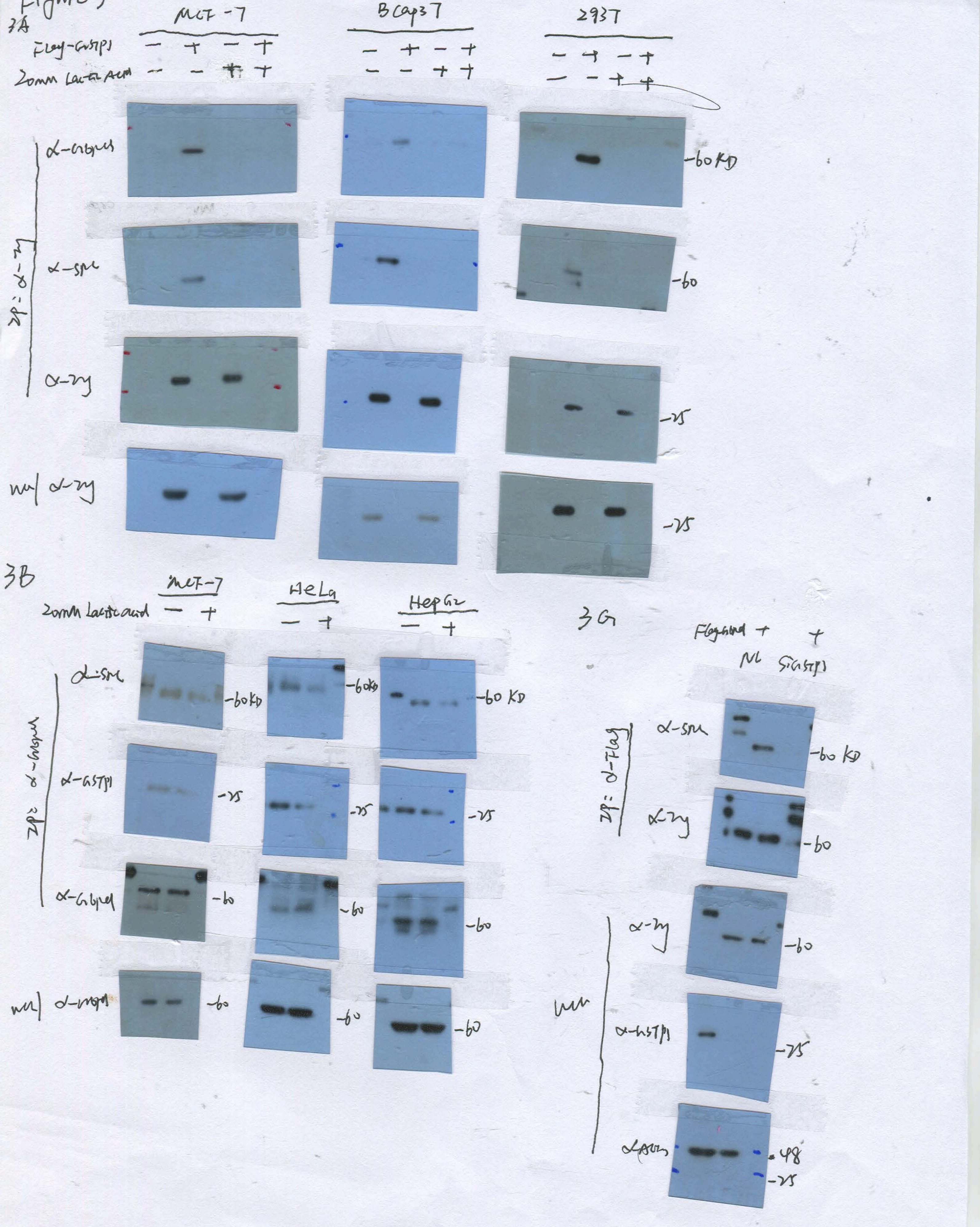

3A
MCF-7
Bcap37
293T
-60 KD
-60
-25
3B
MCF-7
HeLa
HepG2
-60 KD
-25
-60
3G
NC
IP: α-Flag
-60 KD
-60
-25
-48

Figure3

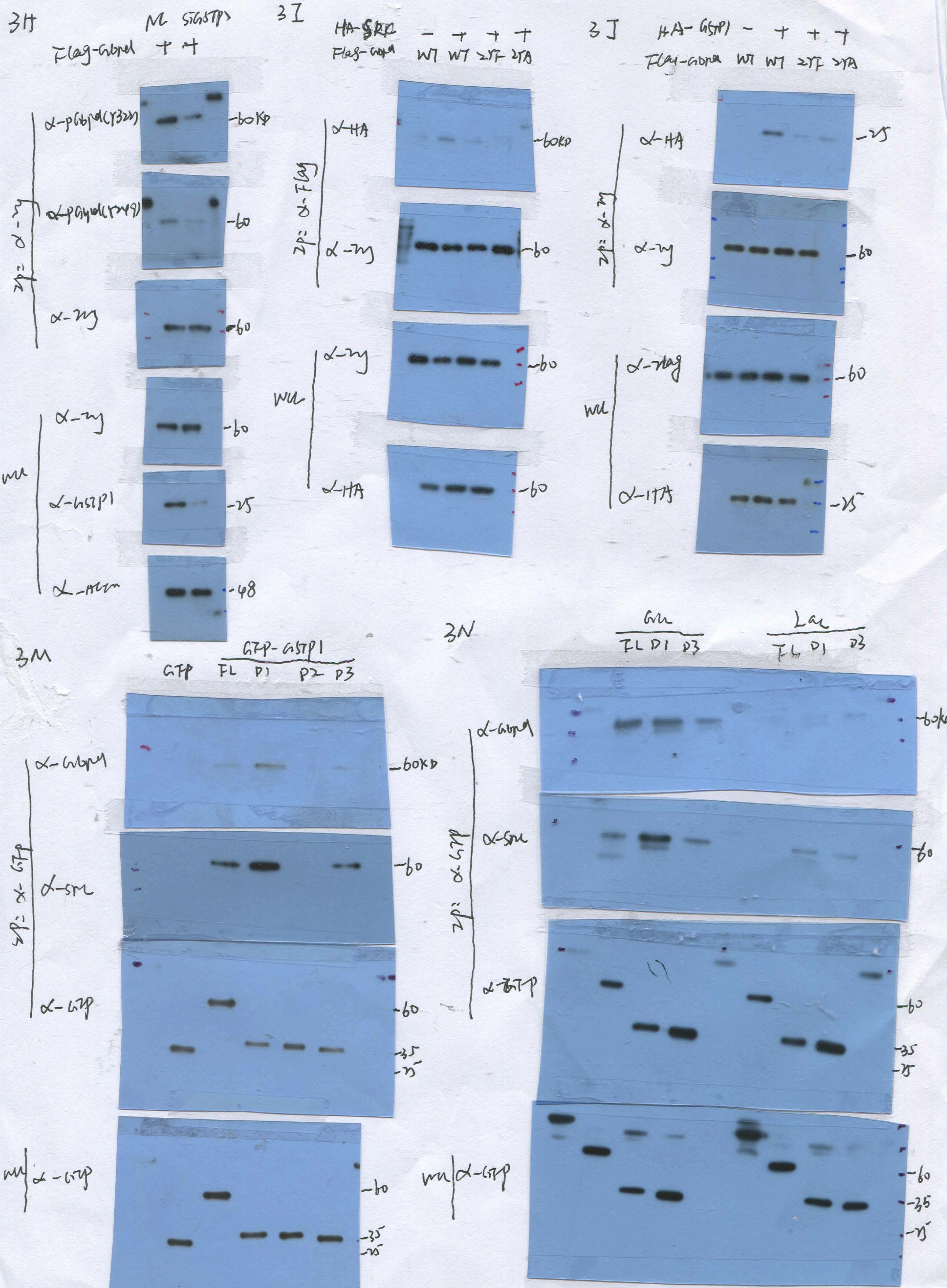

Figure 4

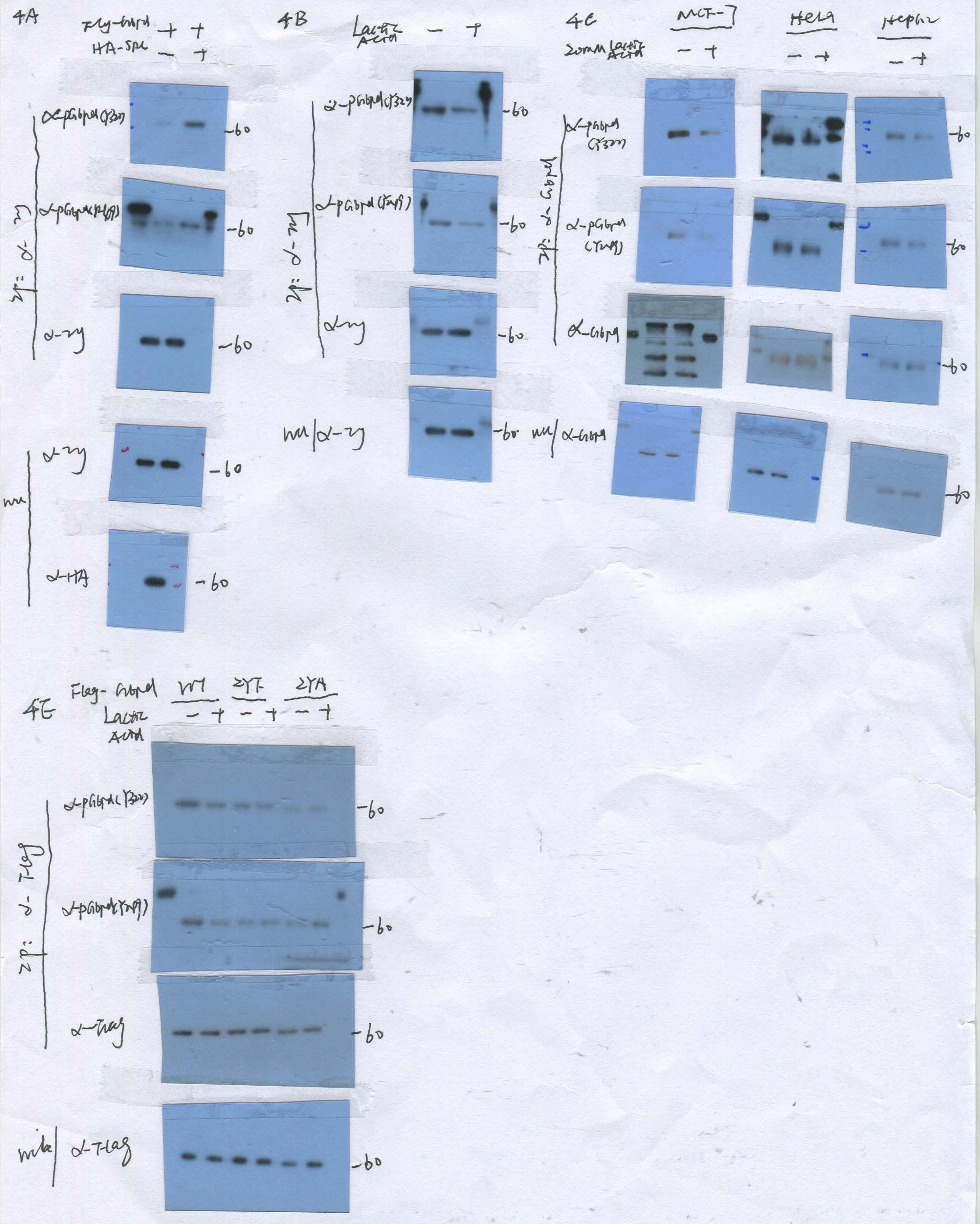
4A
HA-SPL
α-HA
-60
4B
4C
MCF-7
HeLa
HepG2
4E
Flag-G6PD
WT
2YF
2YA
α-Flag

Figure 5

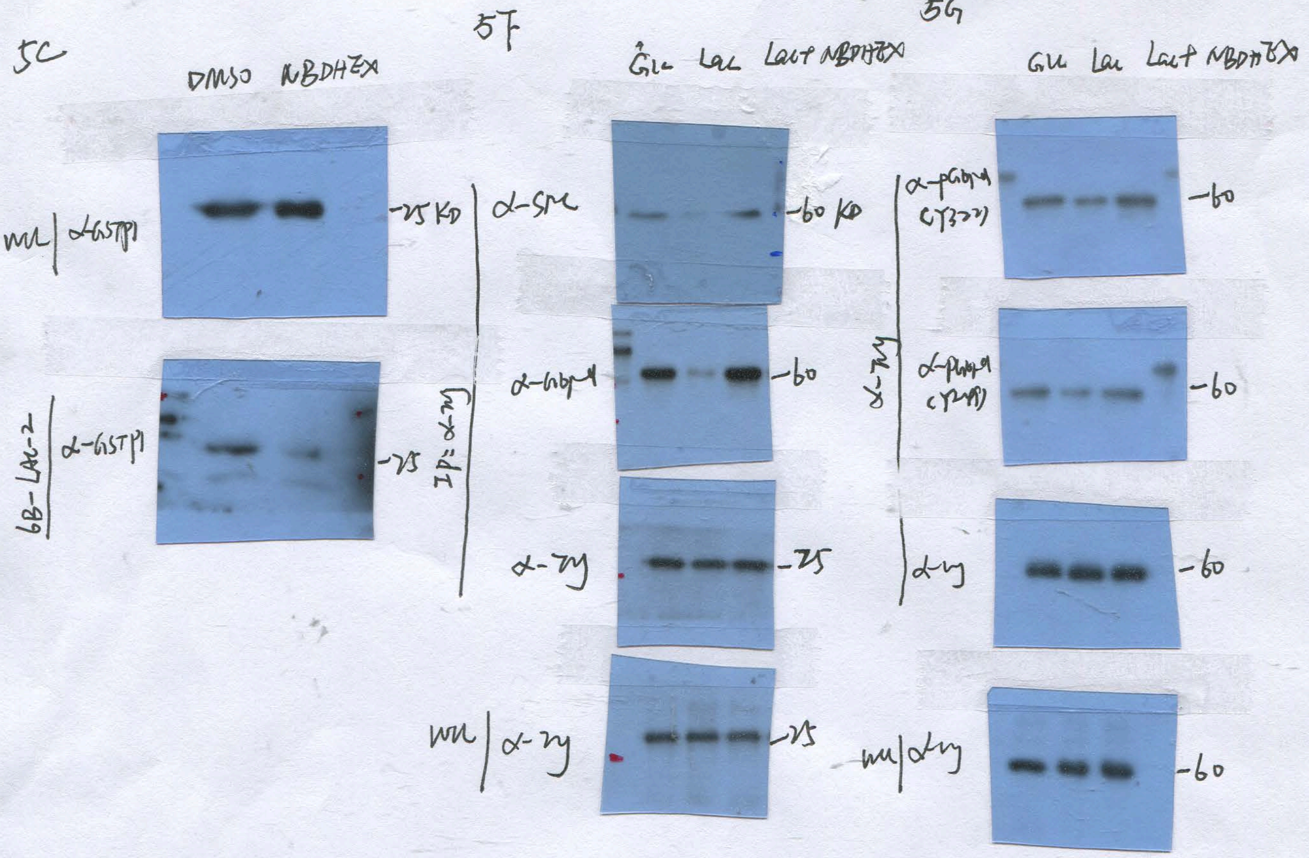

5C
DMSO NBDHEX
α-GSTP1
-25 KD
α-GSTP1
-25
5F
-60 KD
-60
-25
-25
5G
-60
-60
-60
-60

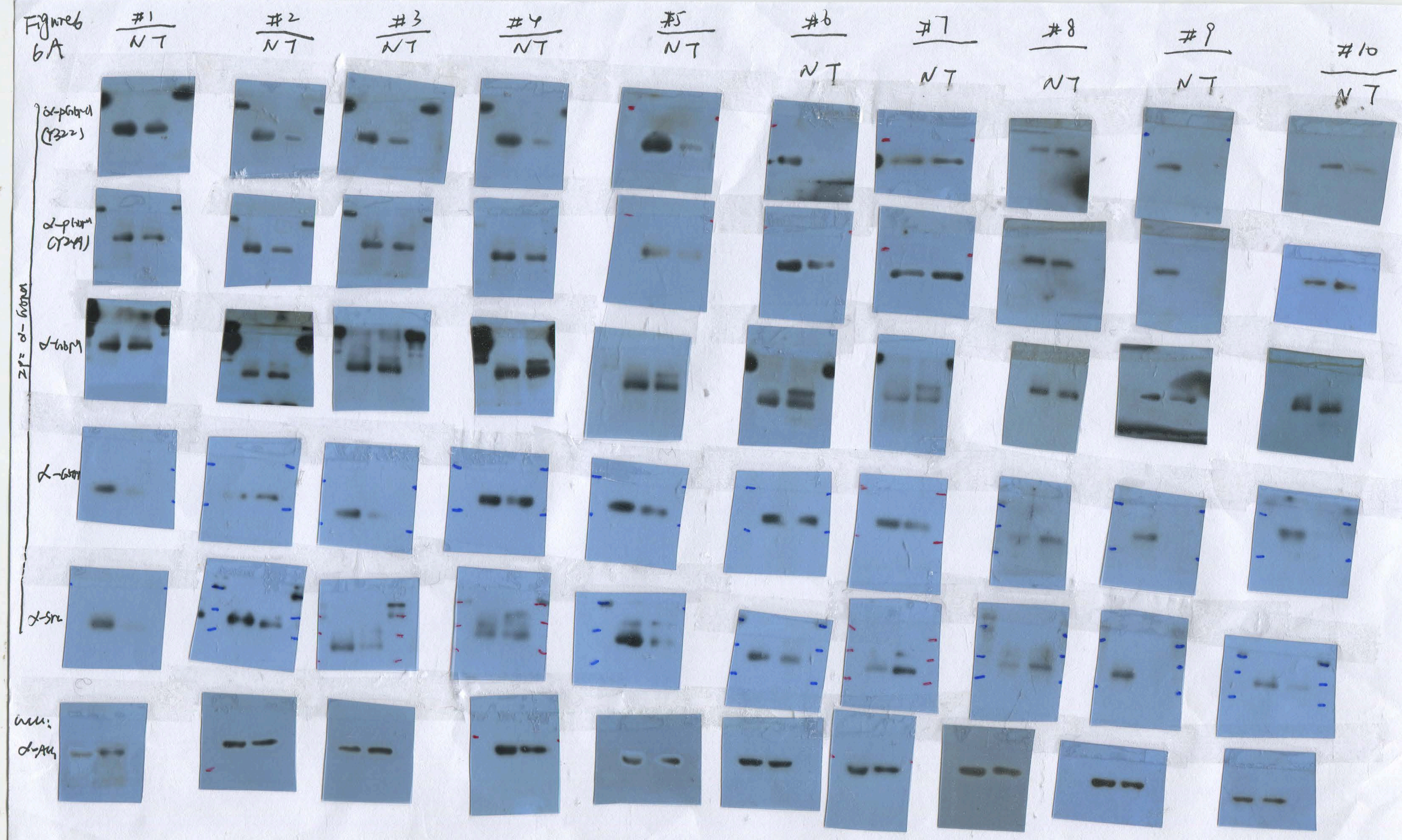

Figure6
6A
#1 N T
#2 N T
#3 N T
#4 N T
#5 N T
#6 N T
#7 N T
#8 N T
#9 N T
#10 N T
(Y322)
α-Src

Figure 6

6A

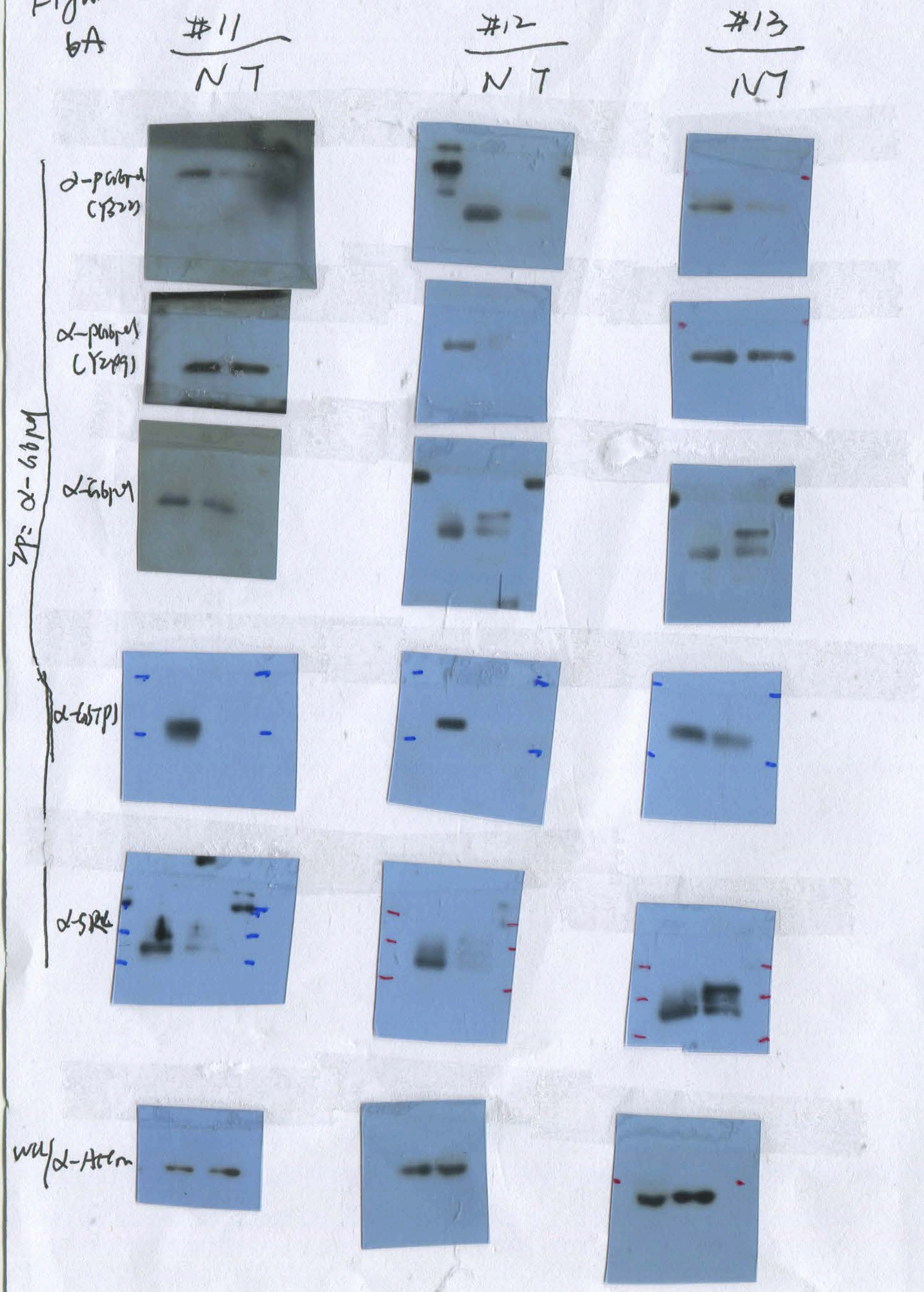

Supplement: Supplementary file 12 — raw WB data [file 41419_2023_5998_MOESM12_ESM.pdf]
