# Supplementary material for: A GSTP1-mediated lactic acid signaling promotes tumorigenesis through the PPP oxidative branch
Source: Cell Death Dis. 2023 Jul 25;14(7):463. doi: 10.1038/s41419-023-05998-4 (PMC10368634; doi:10.1038/s41419-023-05998-4)
Supplement: Supplementary file 13 — raw supplementary data [file 41419_2023_5998_MOESM13_ESM.pdf]

Figure S1

S1 F

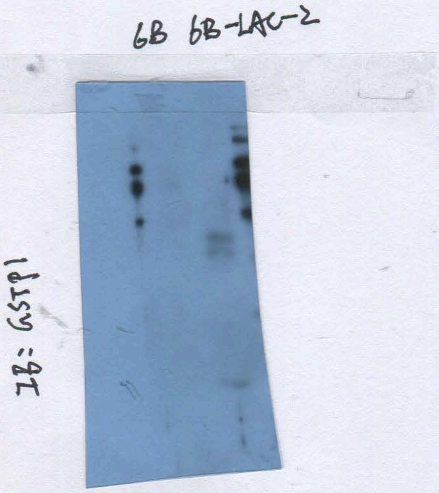

Figure S2

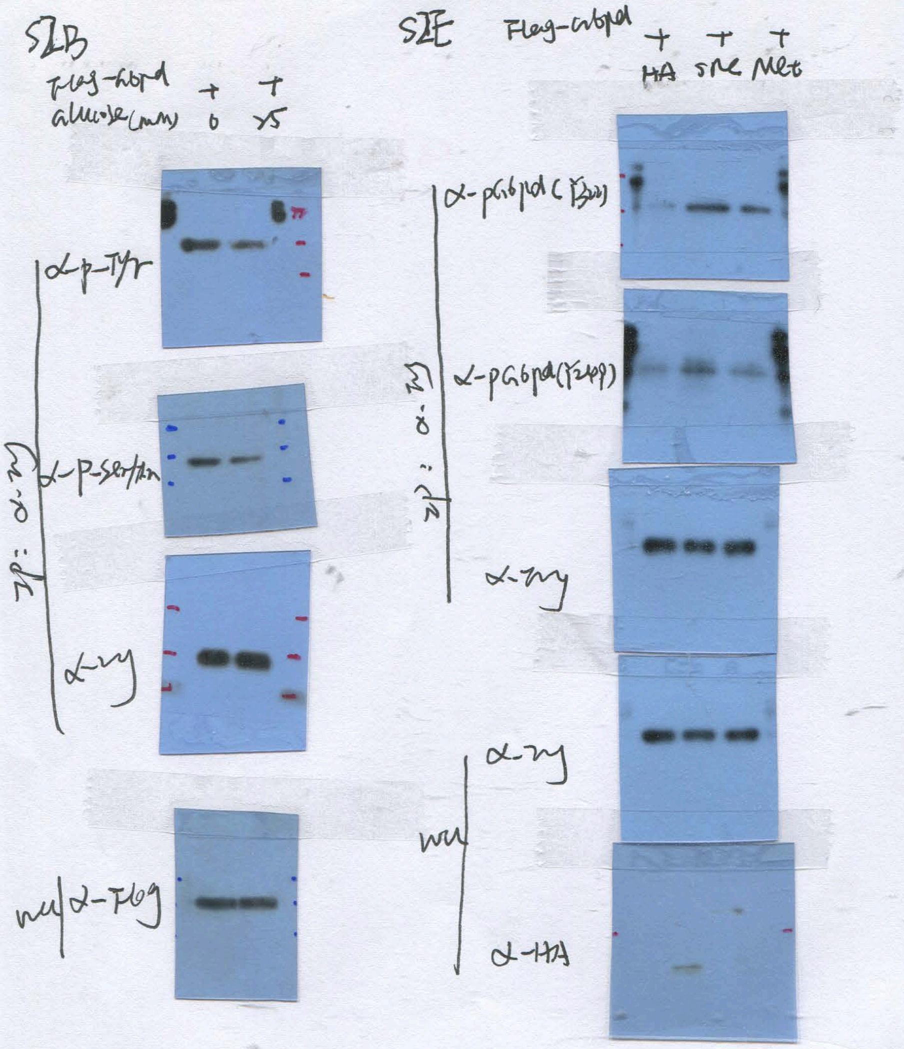

Figure S3

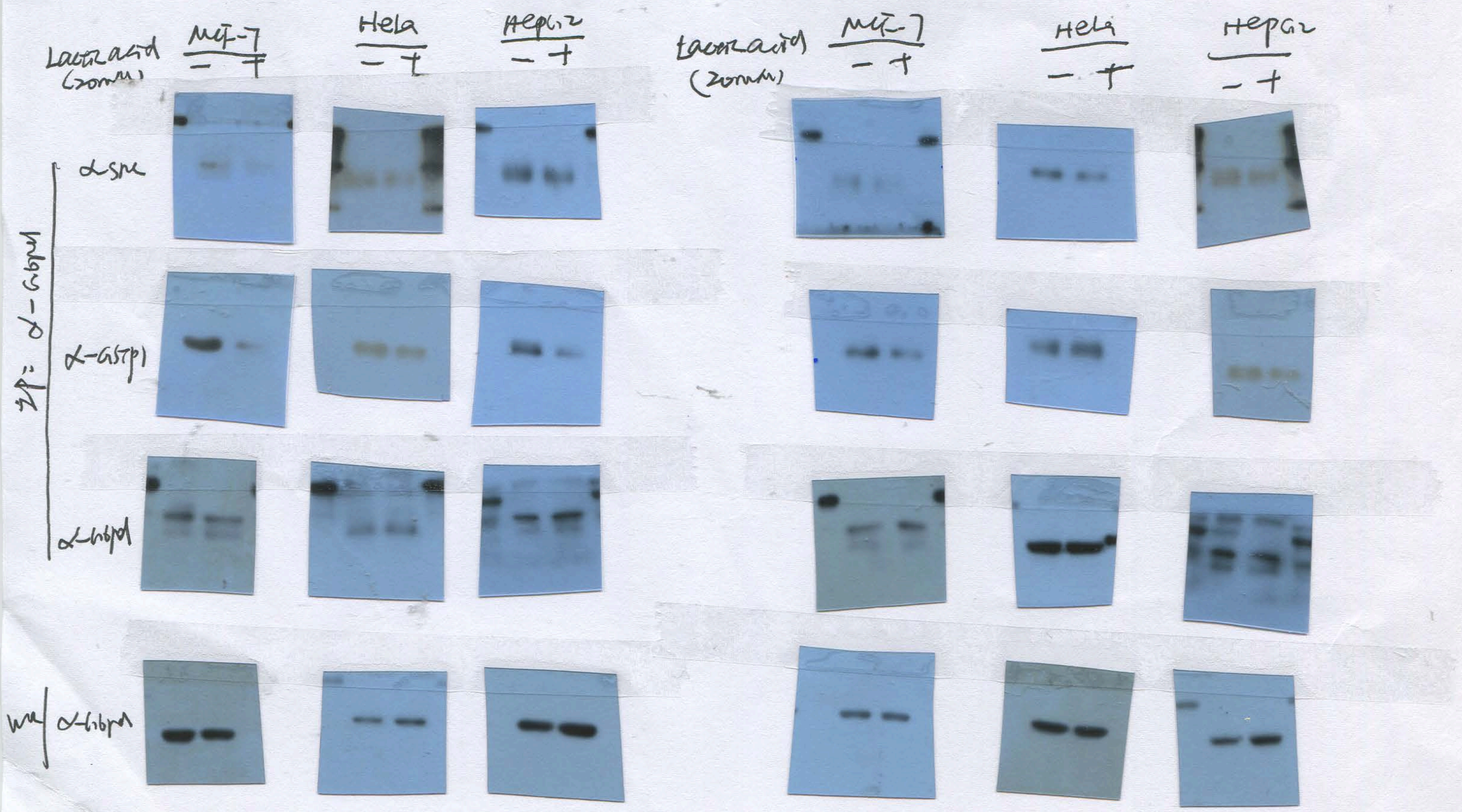

Figure S4

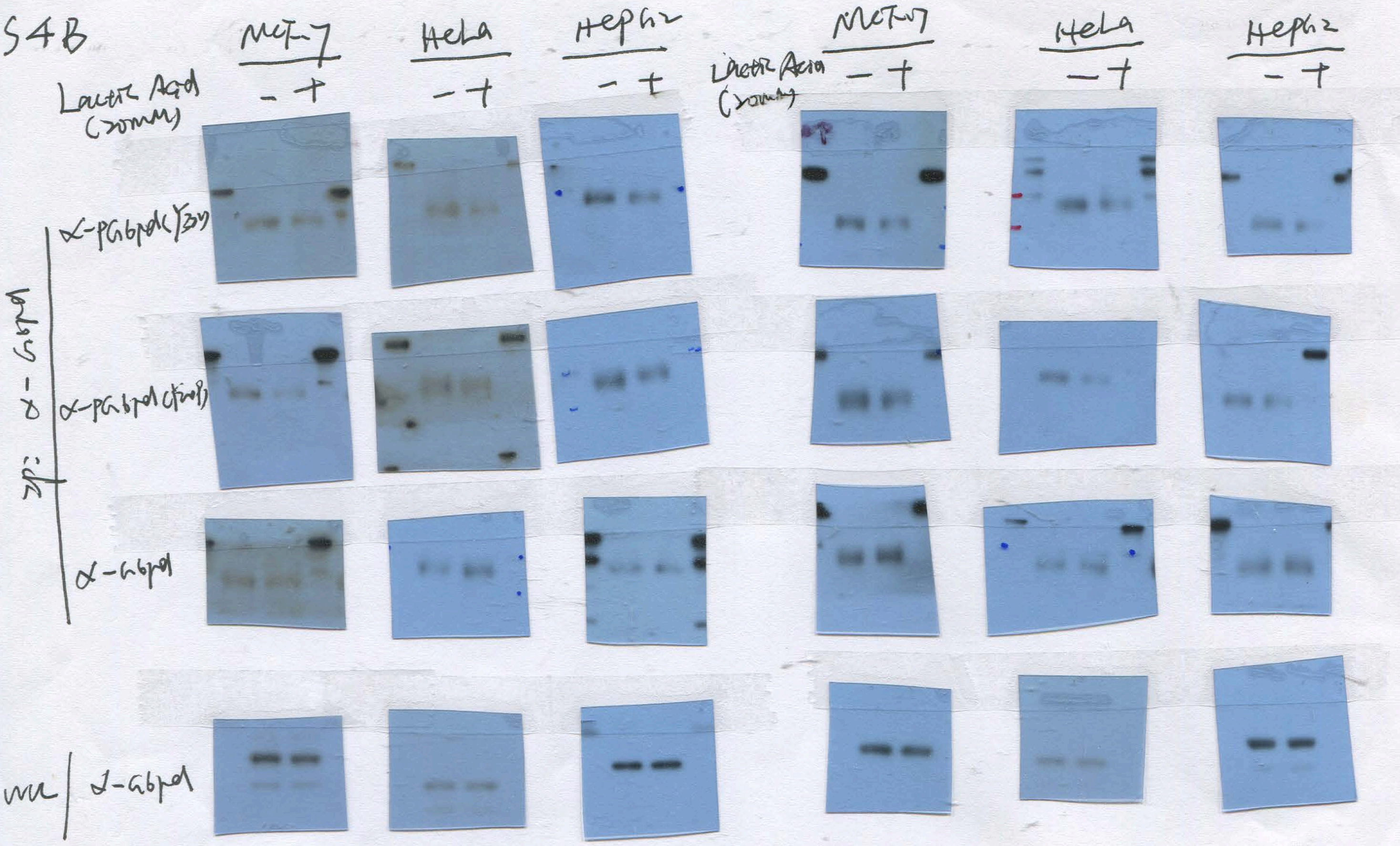

Figure S5

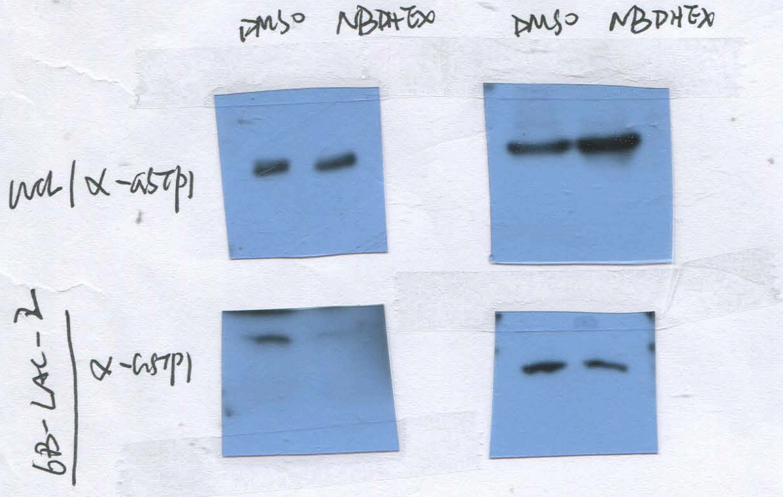

Figure S7

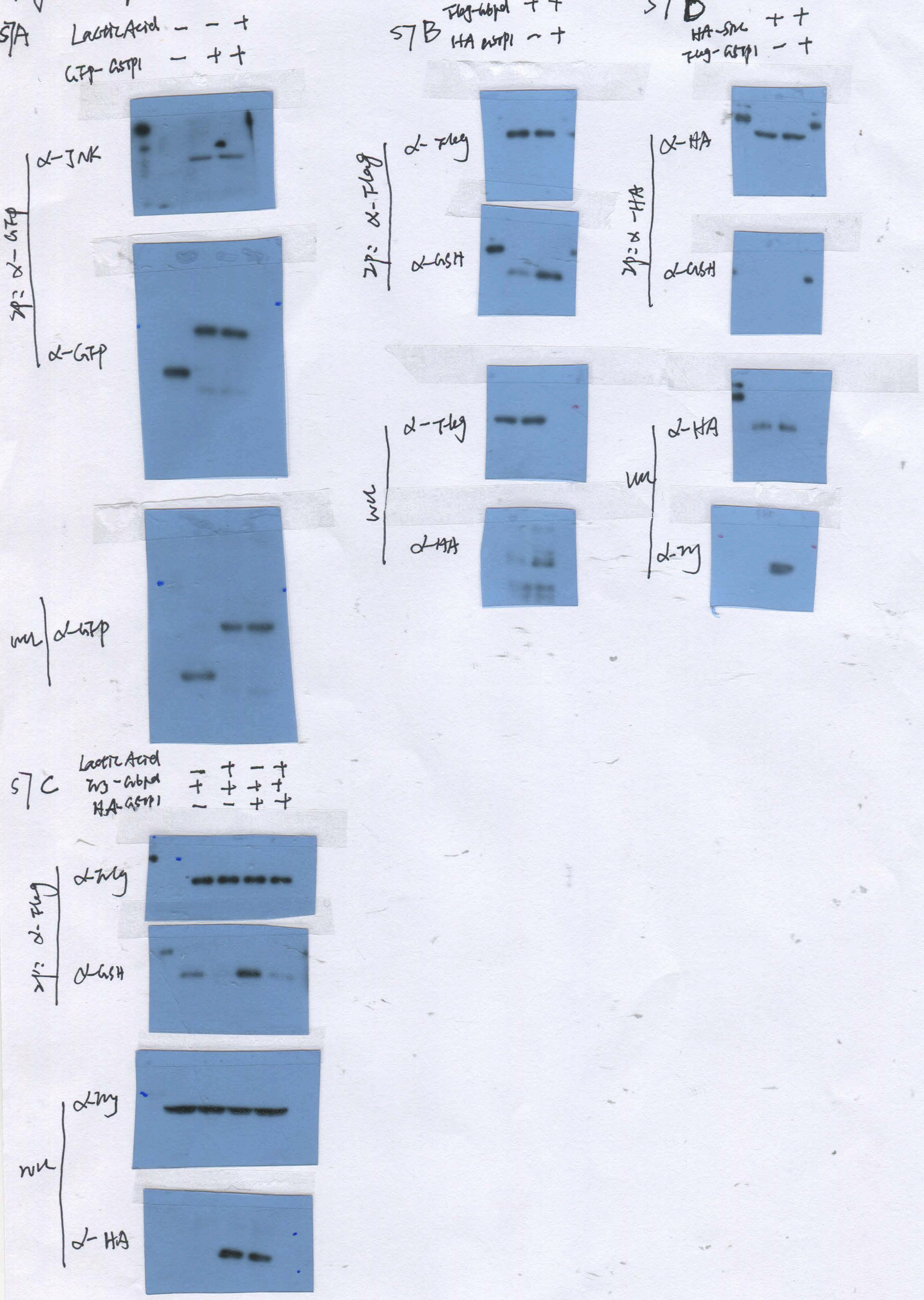
S7A
Lactic Acid - - +
GFP-GSTP1 - + +
IP: α-GFP
α-JNK
α-GFP
WCL
α-GFP
S7B
HA GSTP1 - +
IP: α-Flag
α-Flag
α-GSH
WCL
α-Flag
α-HA
S7D
Flag-GSTP1 - +
IP: α-HA
α-HA
α-GSH
WCL
α-HA
S7C
Lactic Acid - + - +
HA-GSTP1 - - + +
IP: α-Flag
α-GSH
WCL
α-HA
